# Metabolic modelling reveals the specialization of secondary replicons for niche adaptation in *Sinorhizobium meliloti*

**DOI:** 10.1038/ncomms12219

**Published:** 2016-07-22

**Authors:** George C. diCenzo, Alice Checcucci, Marco Bazzicalupo, Alessio Mengoni, Carlo Viti, Lukasz Dziewit, Turlough M. Finan, Marco Galardini, Marco Fondi

**Affiliations:** 1 Department of Biology, McMaster University, Hamilton, Ontario, Canada L8S 1A1; 2 Department of Biology, University of Florence, 50019 Sesto Fiorentino, Italy; 3 Department of Agri-food Production and Environmental Sciences, University of Florence, 50144 Sesto Fiorentino, Italy; 4 Department of Bacterial Genetics, Institute of Microbiology, Faculty of Biology, University of Warsaw, 02-096 Warsaw, Poland; 5 EMBL-EBI, Wellcome Trust Genome Campus, Cambridge CB10 1SD, UK

## Abstract

The genome of about 10% of bacterial species is divided among two or more large chromosome-sized replicons. The contribution of each replicon to the microbial life cycle (for example, environmental adaptations and/or niche switching) remains unclear. Here we report a genome-scale metabolic model of the legume symbiont *Sinorhizobium meliloti* that is integrated with carbon utilization data for 1,500 genes with 192 carbon substrates. Growth of *S. meliloti* is modelled in three ecological niches (bulk soil, rhizosphere and nodule) with a focus on the role of each of its three replicons. We observe clear metabolic differences during growth in the tested ecological niches and an overall reprogramming following niche switching. *In silico* examination of the inferred fitness of gene deletion mutants suggests that secondary replicons evolved to fulfil a specialized function, particularly host-associated niche adaptation. Thus, genes on secondary replicons might potentially be manipulated to promote or suppress host interactions for biotechnological purposes.

## Abstract (web summary)

The genome of some bacteria consists of two or more chromosomes or replicons. Here, diCenzo *et al*. integrate genome-scale metabolic modelling and growth data from a collection of mutants of the plant symbiont *Sinorhizobium meliloti* to estimate the fitness contribution of each replicon in three environments.

## Graphical abstract

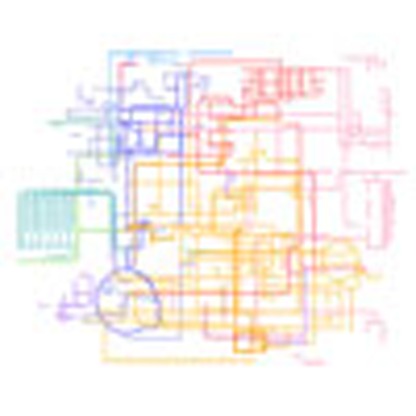

The last years have witnessed a growing attention towards the ecological and evolutionary implication of the multiple replicon bacterial genome^1^,^2^,^3^,^4^ that is present in about 10% of sequenced bacterial genomes^1^. This genome architecture is common in the proteobacterial species that interact with a host and are of importance to the human population^1^,^2^, including crop plant symbionts (for example, *Sinorhizobium* and *Rhizobium*), plant pathogens (for example, *Agrobacterium*), and animal and human pathogens (for example, *Brucella*, *Burkholderia* and *Vibrio*). As the bacterial genome is non-randomly organized^5^, it is proposed that this genome organization was shaped by selective pressures to facilitate improved host interactions and niche adaptation. Though it is well established that secondary replicons often carry genetic determinants essential to colonize a novel environment, for example, virulence or symbiotic genes, such genes often account for only a small proportion of these replicons^6^,^7^. The majority of the genes on a secondary replicon are not directly essential to colonize a specific environment, and the adaptive function of these genes and why they are localized on a secondary replicon remains unclear. Several recent studies have provided evidence consistent with the secondary replicons in a multipartite genome encoding environment-specific fitness promoting but non-essential functions^3^,^4^,^8^,^9^,^10^,^11^,^12^. However, none of these studies demonstrated that secondary replicons indeed carry environment-specific fitness determinants, thus serving as reservoirs for niche-specific functions.

*Sinorhizobium meliloti* is a N_2_-fixing endosymbiont of legume species that has recently become a model organism for the study of bacterial multipartite genome function and evolution. All sequenced *S. meliloti* genomes contain at least three large replicons (the primary chromosome, the pSymB chromid and the pSymA megaplasmid), with some strains hosting additional small accessory plasmids^3^,^13^,^14^. *S. meliloti* experiences a complex life cycle and successfully colonizes three distinct niches. Two of these niches are bulk soil and rhizosphere soil (that is, the soil directly influenced by the plant root system), which are quite different environments, with the rhizosphere generally considered to be a nutritionally richer environment due to plant root exudates^15^. The third niche inhabited by *S. meliloti* is the legume root nodule. *S. meliloti* can induce root nodule formation in certain legumes and within nodules the bacteria differentiate into N_2_-fixing bacteroids. Manipulation and optimization of this agriculturally and ecologically important symbiosis is an ultimate goal of the rhizobial research community^16^,^17^,^18^. Effectively doing so will require a complete understanding of the evolution^18^, genetics^6^ and the metabolism of the organism in both rhizosphere and nodule environments, as well as the corresponding metabolic shifts.

Here we combine a genome-scale metabolic network reconstruction of the *S. meliloti* genome, flux balance analysis (FBA), and growth phenotype data for 11 large-scale *S. meliloti* deletion mutants to examine the metabolic changes accompanying the shifts between bulk soil, rhizosphere and nodule environments. We use an *in silico* approach to predict the phenotypes resulting from the deletion of 1,575 *S. meliloti* metabolic genes, estimate the fitness contribution of each replicon within each environment, and thus provide insight into the evolution of multipartite genomes.

## Results

### Reconstruction of a *S. meliloti* genome-scale metabolic model

As described in the Supplementary Methods, an *in silico* representation of the metabolism of *S. meliloti* was developed, and the final model that was termed iGD1575 contained 1,575 genes, 1,825 reactions and 1,579 metabolites. iGD1575 accounts for 25.4% of the protein-coding genes in the *S. meliloti* genome, and the other main features of the model are listed in Table 1. Cluster of Orthologous Gene (COG) analyses confirmed that the gene functional biases of each replicon are accurately represented in iGD1575 (Supplementary Fig. 1)^12^,^13^. The iGD1575 model encompasses 529 of the 565 genes present in iHZ565, a previously reported *S. meliloti* small metabolic model^19^. The remaining 31 genes were not added to iGD1575 as experimental data were inconsistent with their annotation, we felt their annotation too general to have high confidence in the enzymes' substrates/products, or the associated reaction involved a metabolite not present in any other reaction in the model and thus the reaction would never carry flux in FBA (Supplementary Table 1). Comparison of the number of genes in iGD1575 to that of other available rhizobial and non-rhizobial models^19^,^20^,^21^,^22^ showed that iGD1575 is currently one of the largest metabolic reconstruction of a bacterial genome. In addition, iGD1575 is the first metabolic model capable of representing the metabolism of both a symbiotic and free-living rhizobial cell.

### Quantitative validation of iGD1575

Previous work^23^ has shown that *S. meliloti* transports glucose into the cell at a rate of 2.41 mmol h^−1^ per g cellular dry weight. When glucose is provided to the iGD1575 model as the sole carbon source at the experimentally determined rate, a specific growth rate of 0.325 h^−1^ is predicted, which is consistent with our experimentally derived growth rate of 0.313 h^−1^ (s.d. 0.002) for *S. meliloti* grown with glucose. Similarly, it has been shown^24^ that *S. meliloti* transports succinate into the cell at a rate of 6.252 mmol h^−1^ per g protein. Providing succinate as the sole source of carbon to iGD1575 at the experimentally determined value led to a predicted specific growth rate of 0.279 h^−1^, similar to the experimentally derived growth rate of 0.254 h^−1^ (s.d. 0.025) for *S. meliloti* grown with succinate. Measuring the amount of phosphate remaining in the spent growth media following growth of *S. meliloti* indicated that 63.7 (s.d. 6.7) and 39.8 μM (s.d. 1.5) of phosphate was used per mM of glucose and succinate, respectively. These experimental values are relatively consistent with the phosphate usage values predicted by iGD1575 of 72.7 and 48.5 μM per mM of glucose and succinate, respectively.

Little experimental flux data has been reported for *S. meliloti*; however, flux measurements for 22 central carbon metabolic reactions when *S. meliloti* is grown with glucose as the sole source of carbon have been reported by Fuhrer *et al*.^23^. Not surprisingly, the experimentally determined fluxes did not match well with the iGD1575-derived values. This is because the specific growth rate of *S. meliloti* was only 0.17 h^−1^ in the study by Fuhrer *et al*., indicating that *S. meliloti* was grown in sub-optimal conditions that presumably effected the flux distribution. Nevertheless, if the flux through these 22 central carbon metabolic reactions in iGD1575 was set to that as experimentally determined by Fuhrer *et al*., the predicted specific growth rate was reduced to 0.159 h^−1^, in line with the 0.17 h^−1^ reported by Fuhrer *et al*. The good relationship between flux distribution and specific growth rate, and the strong ability of iGD1575 to predict growth rate and phosphate usage when grown with glucose or succinate, suggest that the flux distributions predicted by iGD1575 should represent quantitatively accurate estimations.

### iGD1575 captures the metabolic capacity of *S. meliloti*

**Figure 1:**
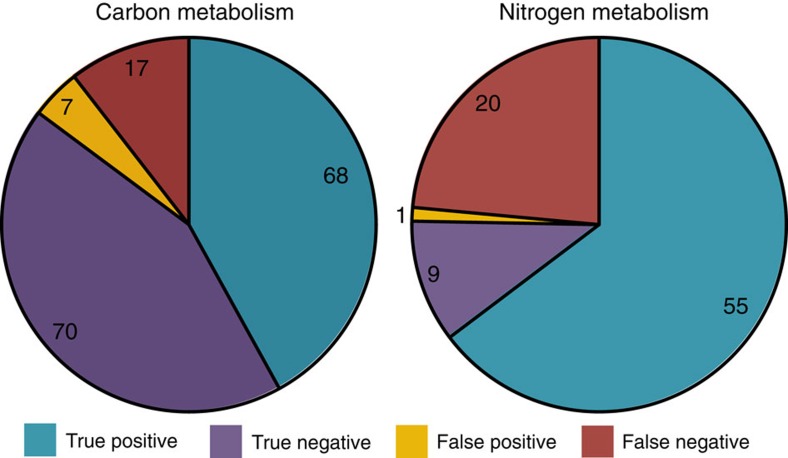
Agreement between experimental and *in silico* metabolic capabilities of *S. meliloti*. True positives, growth was observed experimentally and *in silico*. True negatives, growth was not observed experimentally or *in silico*. False negatives, compounds that support growth experimentally but not *in silico*. False positives, compounds that support growth *in silico* but not experimentally. The complete set of compounds and growth predictions can be found in Supplementary Data 1.

The ability of *S. meliloti* to grow with various carbon and nitrogen sources has been well studied by means of the Phenotype MicroArray (Biolog) technology^4^,^25^,^26^,^27^. These previously published studies were used to guide model expansion and refinement during the curation process. Once all of the manual curation of iGD1575 was complete, FBA illustrated that the final model could accurately predict the ability of *S. meliloti* to produce, or not produce, biomass (as defined in Supplementary Table 2) on 85% (138/162) and 75% (64/85) of the tested carbon and nitrogen substrates, respectively, for which the ability of *S. meliloti* to utilize, or not, these compounds is known (Fig. 1 and Supplementary Data 1). Most of the discrepancies between the experimental data and the iGD1575 growth prediction were false negatives (71% and 95% for growth with carbon and nitrogen substrates, respectively). These represent compounds that *S. meliloti* can metabolize but the model cannot use for the production of biomass, likely representing gene annotation gaps in our knowledge of *S. meliloti* that will serve as targets for future research. The predictive power of bacterial metabolic models reported in previous studies^28^,^29^,^30^ is similar to that reported here for iGD1575. Hence, iGD1575 is at least as good as other current genome-scale metabolic reconstructions at representing the organism's metabolic capabilities. This suggests that iGD1575 effectively captures the metabolic capacity of *S. meliloti* and can validly be used to model metabolism in nutritionally diverse environments.

### Carbon growth phenotypes of *S. meliloti* deletion mutants

Carbon utilization phenotypes for a subset of large-scale pSymB deletion mutants^31^ that cumulatively remove ∼1.65 Mb (98%) of pSymB (Supplementary Fig. 2) were determined using PM1 and PM2A Biolog plates. This screen effectively generated a carbon utilization data set for ∼1,500 pSymB genes. Overall, growth was observed with 76 carbon substrates, and a total of 43 no or extremely poor growth phenotypes were observed (Table 2, Supplementary Figs 3 and 4, and Supplementary Data 1 and 2). In the process of developing and validating iGD1575, an *in silico* representation of the same experiment was performed, and where possible, the model was updated to fix discrepancies between the experimental and *in silico* results. Following this integration of the Phenotype MicroArray data set with the metabolic reconstruction, there was very good agreement between the experimentally observed results and the *in silico* simulations was observed (Table 2 and Supplementary Data 1). *In silico* simulations did not predict any ‘no growth' phenotypes that were not experimentally observed, and 23 of the 36 (63.9%) experimentally observed phenotypes for compounds that support growth of iGD1575 were replicated *in silico*. Some of the discrepancies between the experimental and *in silico* data represent gaps in our knowledge of catabolic pathways in *S. meliloti*, while other phenotypes may occur for non-metabolic reasons and therefore not give a phenotype *in silico*. For example, the *S. meliloti* deletion mutant ΔB154 is more sensitive to cobalt chelation than the wild type^32^, and the lack of growth in wells with L-histidine or D-glucosamine may simply reflect cobalt chelation^33^,^34^.

In addition to model refinement, integrating the mutant phenotype data with iGD1575, the DuctApe software^27^, the *S. meliloti* genome annotation^13^ and an ABC transporter induction study^35^ allowed for the prediction of novel carbon catabolic loci. One example compound is the monosaccharide psicose. Our analysis suggests that psicose is transported by the SupABCD (Smb20484–Smb20487) ABC transporter, and then converted to fructose by an isomerase encoded by *smb20488*. A second example is D-galactosamine, which, as elaborated on in Supplementary Note 1, we predict is transported by the Smb21216, Smb21219–Smb21221 transporter and potentially the Smb21135–Smb21138 transporter, and then metabolized by Smb21217, Smb21218, Smb21373 and Smb21374.

### Rhizosphere colonization required a metabolic refinement

**Figure 2:**
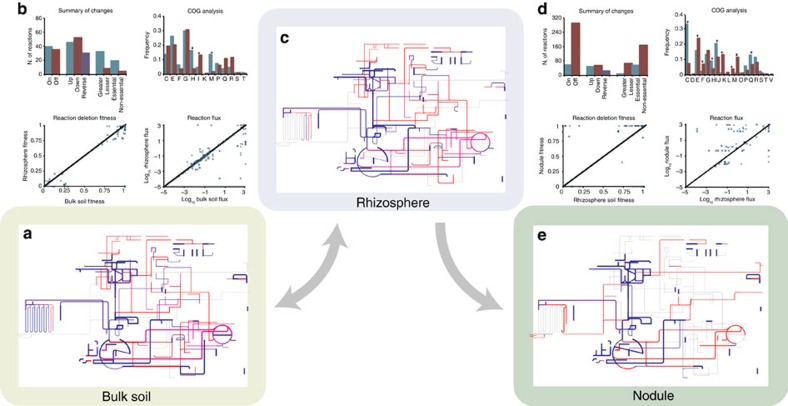
The effect of niche conditions on the reconstructed metabolic network. Networks were visualized following optimization in (**a**) bulk soil, (**c**) rhizosphere and (**e**) nodule environments. Lines are colour coded based on fitness effect of deleting each reaction: blue indicates a fitness decrease <1%; dark purple indicates a fitness decrease <50%; bright purple indicates a fitness decrease >50%; and red indicates a fitness decrease >99%. Thin grey lines indicate inactive reactions. Line thickness shows the flux through each reaction on a log scale. The graphs summarize the metabolic changes detected during the (**b**) bulk soil to rhizosphere and (**d**) rhizosphere to nodule transitions. Summary of changes graphs: on and off—reactions carrying flux only in the second and first environment, respectively; up and down—reactions carrying increased flux (≥50%) in the second and first environment, respectively; reverse—reactions whose directionality is switched; greater and lesser—reactions whose removal have a greater (≥10%) fitness impact in the second and first environment, respectively; essential and non-essential—reactions essential only in the second and first environment, respectively. The nine classifications are not mutually exclusive. The reactions present in each category are described in Supplementary Data 3 and 4. The COG analysis graphs summarize the functional annotation of the genes associated with the reactions in the summary of changes graphs. The blue and red bars include the genes associated with the blue and red bars, respectively, in the summary of changes graphs. Asterisks indicate statistically significant changes (*P* value <0.01) as determined by Pearson's *χ*^2^-tests. In the reaction flux figures, each point represents the amount of flux through individual reactions in the two environments. Blue and purple symbols indicate reactions with the same or reverse directionality, respectively. The angled line indicates the position of a perfect correlation. In the reaction deletion fitness figures, each point represents the fitness of individual reaction deletion mutants in the two environments. The angled line indicates the position of a perfect correlation.

The metabolic shifts experienced by *S. meliloti* during transition between bulk soil, the rhizosphere and the nodule were modelled using *in silico* representations of the nutritional composition of each environment. These took into account the relative ratios of each component in the different environments and the development of these environments are described in the Supplementary Methods. In the bulk soil and rhizosphere environments, the model was optimized for the production of biomass as defined in Supplementary Table 2, whereas in the nodule environment the model was optimized for production of an effective N_2_-fixing symbiosis as defined previously^19^. The optimal flux patterns in each of the three niches were obtained using FBA and visualized with iPath (Fig. 2)^36^.

The metabolic network appears globally similar in both the bulk soil and rhizosphere environments (Fig. 2 and Table 3), although many subtle differences were present when reaction specific parameters were examined (Fig. 2 and Supplementary Data 3). Despite good correlation between the log_10_ of the absolute flux through a given reaction that was active in both environments (*P* value <0.01 using a Spearman's Rank Order Correlation test, median (absolute residual/observed)=0.09; Supplementary Fig. 5a), ∼20% of the reactions showed at least a 50% change in flux between the two environments while an additional 6% switched directions. Similarly, the effect on fitness (defined as the flux through the objective function (biomass formation or symbiosis) in the mutant relative to the flux through the objective function in the wild type) of individual reaction deletions displayed a strong correlation between the two environments (*P* value<0.01 using a Spearman's Rank Order Correlation test, *R*^2^=0.95; Supplementary Fig. 5b). Nevertheless, ∼7% had at least a 10% variation in fitness effect between environments, and ∼4% were essential in just one of the two niches. Interestingly, optimal growth in the rhizosphere required a greater repertoire of metabolic reaction as illustrated by the increased number of reactions required for maximal fitness. In addition, ∼13% of the active reactions were specific to just one of the environments. The reactions whose fluxes were considered to change between growth in bulk soil and the rhizosphere were further validated through a procedure involving flux variability analysis as detailed in Supplementary Note 2.

Few outstanding biases (*P* value <0.01 using a Pearson's *χ*^2^-test) were seen in the COG annotations of the genes associated with reactions whose flux or fitness contribution was dependent on the soil environment. This indicated that the reactions important in the rhizosphere were biologically similar to, but functionally distinct from, the reactions important in bulk soil. However, coenzyme transport and metabolism (COG H), and cell wall, membrane and envelope biogenesis (COG M) were more important in the rhizosphere than in the bulk soil. This possibly reflects different coenzyme requirements for the metabolic pathways active in the two environments and the increased succinoglycan content of *S. meliloti* in the rhizosphere that is necessary to facilitate root biofilm formation. Lipid transport and metabolism (COG I) was over-represented in the bulk soil, perhaps due to the over-abundance of ketogenic amino acids in bulk soil. At the pathway level, only a few changes could not be explained by differences in the nutritional composition and biomass objective functions (Supplementary Data 3). For example, the importance of various carbon catabolic pathways and amino-acid biosynthetic pathways reflected the abundance of the sugars and amino acids in each environment. This analysis also revealed that *S. meliloti* relies more heavily on glycolytic substrate during growth in bulk soil but on gluconeogenic substrate in the rhizosphere, which was consistent with the high concentration of organic acids in the rhizosphere. The increased gluconeogenic flux and the increased flux through the pantothenate and coenzyme-A biosynthesis pathways in the rhizosphere is also consistent with an increased sugar demand for the rhizosphere-specific Nod factor production and increased exopolysaccharide biosynthesis^37^,^38^. Finally, the urea cycle contributed more to cellular fitness in bulk soil than in the rhizosphere.

### Complex metabolic reprogramming is associated with symbiosis

The rhizosphere to nodule transition was accompanied with much more pronounced metabolic changes than the bulk soil to rhizosphere transition (Fig. 2 and Supplementary Data 4). Half as many reactions carried flux in the nodule than in the rhizosphere, with ∼61% of rhizosphere reactions off in the nodule and ∼22% of active nodule reactions off in the rhizosphere. This overall decrease in metabolic reactions active in the nodule is consistent with the global transcriptional downregulation in differentiated bacteroids^39^,^40^. For reactions active in both environments, there was a significant correlation (*P* value<0.01 using a Spearman's Rank Order Correlation test; Supplementary Fig. 5c) in the log_10_ of the absolute flux values, but the dispersion of the observed values from the regression line was high (median (residual/observed)=1.48). Approximately half of the common flux carrying reactions displayed at least 50% more flux in one of the environments and a further 12% switched directions. In addition, little correlation was observed between the fitness effects of individual reaction deletions in the two environments (*R*^2^=0.03; Supplementary Fig. 5d). Of the active reactions, ∼38% were essential specifically in one environment, while the deletion of another 12% gave fitness effects ≥10% different in the two niches. The reactions whose fluxes were considered to change between growth in the rhizosphere and symbiosis in the nodule were further validated through a procedure involving flux variability analysis as detailed in Supplementary Note 2.

A clear shift in the functional annotation of genes associated with the variable reactions was observed. Functions associated with generating the large amount of energy required for nitrogen fixation displayed increased importance in the nodule: for example, energy production and conversion (COG C), and coenzyme transport and metabolism (COG H). On the other hand, the lack of growth of the differentiated bacteroids not surprisingly rendered biomass component biosynthesis (COGs E, F, L, M, I and J) less important. A few additional interesting observations were noted by looking at pathway level changes (Supplementary Data 4). The Kreb's cycle and AMP synthesis were increased, presumably to accommodate the high ATP demand of nitrogenase. Glycolysis was less important in the nodule, consistent with the lack of glycolysis-specific enzymes detected in the *S. meliloti* nodule proteome^41^. Flux through various pathways producing compounds (including steroids, glutathionine, vitamin B6 and haem) required for a successful symbiosis was observed, and in most cases these changes are supported by previously published proteomic, RNA-seq or induction studies^41^,^42^,^43^. Flux through the non-oxidative pentose phosphate pathway, which is poorly studied in *S. meliloti*^44^, was also increased, consistent with the detection of two enzymes of this pathway in the *S. meliloti* nodule proteome^41^ and the need for *S. meliloti* to synthesize sugars for biosynthesis^45^.

### *S. meliloti* replicons encode niche-specific metabolism

**Figure 3:**
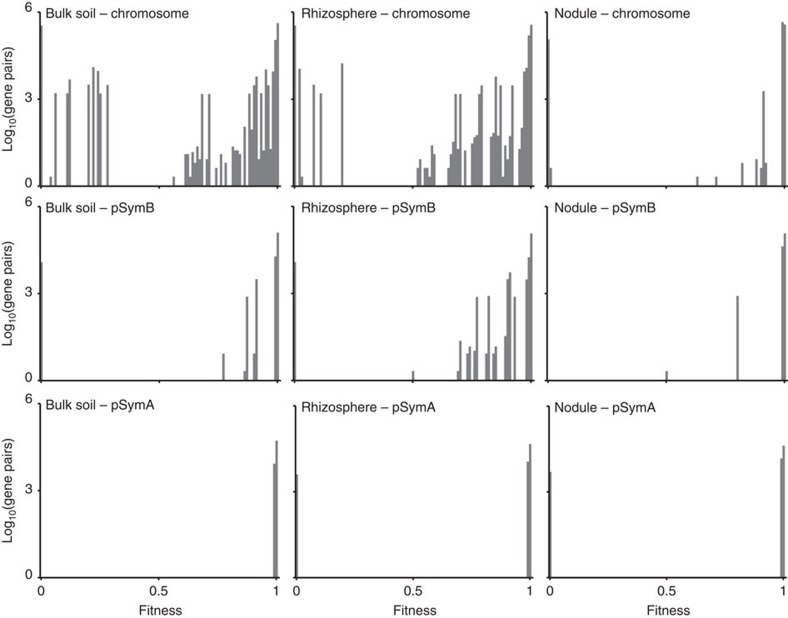
Fitness costs associated with double gene deletions in the three tested ecological niches. All possible pairs of genes present on the same replicon were individually removed from the model, the ability of the resulting mutant to produce flux through the objective function was examined with FBA, and the fitness (solution value of the mutant/solution value of the wild type) of each mutant was calculated. The histograms summarize the calculated fitness values for each mutant in each of the three environments separately for each replicon. The fitness is displayed on the *x* axis, with the number of mutants displaying that fitness level on the *y* axis. The metabolic relevance of a replicon in a particular environment is represented by the number of mutants showing phenotypes between the two extremes (1 and 0); the greater the metabolic relevance, the greater the number of non-extreme phenotypes.

We performed comprehensive, replicon-specific *in silico* single and double gene deletion analyses to determine the contribution of the three *S. meliloti* replicons to the overall fitness of *S. meliloti* in each of the tested environments (Supplementary Table 3, Fig. 3 and Supplementary Fig. 6). The use of a double gene deletion analysis was intended to account for functionally redundancy gene pairs that would mask phenotypes during the single gene deletion analysis^46^,^47^. As before, fitness was determined as the flux through the objective function of the mutant relative to the wild type, with the biomass formation (Supplementary Table 2) being the objective function during growth in bulk soil and the rhizosphere, and N_2_-fixation^19^ being the objective function in the nodule environment.

The mutant analyses revealed that the *S. meliloti* chromosome had a similar contribution to fitness in bulk soil and the rhizosphere; there was little change in the number of essential or fitness-contributing chromosomal genes in these two environments (Fig. 3 and Supplementary Table 3). However, there was a clear reduction in the importance of the chromosome during symbiosis in the nodule, consistent with microarray data showing that chromosomal genes are over-represented amongst the genes that have low expression levels in the symbiotic bacteria relative to the free-living form^11^.

Similar to the chromosome and consistent with the global *S. meliloti* transcriptional downregulation in the nodule^39^, pSymB contributed more or less only to the fitness of the free-living bacterium, with little role detected in the bacteroids (Fig. 3 and Supplementary Table 3). However, unlike the chromosome, pSymB showed a bias in importance between growth in bulk soil and the rhizosphere; the number of fitness-promoting genes was ∼3.5-fold greater in the rhizosphere. Moreover, every pSymB gene that contributed to fitness in bulk soil had a greater fitness contribution in the rhizosphere. This rhizosphere bias was further amplified when considering the origin of fitness-promoting genes. Of the five pSymB genes contributing to fitness in bulk soil, four are involved in arabinose transport or catabolism^48^. All four of these genes have a chromosomal origin and were transferred to pSymB through an inter-replicon translocation event^49^. We therefore detected only a single gene (*smb20201*) contributing to fitness in bulk soil that originated on pSymB. Similarly, transcriptomics work with the pea symbiont, *Rhizobium leguminosarum*, indicated that one of its plasmids (pRL8) is over-represented in genes upregulated specifically in the pea rhizosphere^50^. However, with a few exceptions, the fitness contributions of the pLR8 upregulated genes in bulk soil versus the rhizosphere were not determined.

Even though these data clearly illustrated that the metabolic capabilities encoded by pSymB are either specific or more important for growth in the rhizosphere than bulk soil, we believe that the observed bias is an under-representation of the actual situation. The succinoglycan biosynthetic genes are classified as essential in both the bulk soil and the rhizosphere due to their inclusion in the biomass objective functions; however, they are not truly essential but likely have greater importance in the rhizosphere through facilitating biofilm formation on the legume root. Furthermore, a more complete formulation of the bulk soil and rhizosphere environment may exaggerate the bias. For example, protocatechuate was not included due to a lack of information on its abundance. However, recent work showed that protocatechuate metabolism improved fitness of *R. leguminosarum* in the rhizosphere^51^, and 13 pSymB genes are involved in protocatechuate transport and metabolism^52^,^53^.

In contrast with the other replicons, the pSymA megaplasmid contributed no fitness-promoting genes (Fig. 3 and Supplementary Table 3). No phenotypes were detected in bulk soil, while the ‘essential' genes in the rhizosphere were due to the removal of Nod factor biosynthetic genes. In fact, Nod factor biosynthesis is not essential for growth but is required for the initiation of symbiosis. In the nodule, the essential genes that were identified were required for the synthesis and functioning of the nitrogenase enzyme. The lack of fitness-contributing pSymA genes in the nodule was somewhat surprising, although consistent with published data^6^,^54^, perhaps suggesting that few genes outside of the core symbiotic machinery contribute to the nitrogen fixation process. Indeed, the large rearrangements in the structure of pSymA between wild-type *S. meliloti* nodule isolates^3^ may reflect low selective constraints on the pSymA megaplasmid, and thus explain the low metabolic contribution and importance of pSymA even during the symbiotic interaction.

The biases observed for the importance of each replicon in the different environments were confirmed via random permutations, testing up to 1,000 different nutritional compositions as described in detail in Supplementary Note 3. These environments were created by randomly varying, at each iteration, the maximal allowable uptake of each nutrient with respect to the original value and by also randomly removing two nutrients from the environment. Despite some interesting biological insights being derived from this analysis (Supplementary Note 3 and Supplementary Figs 7 and 8), little variation was seen in the number of essential plus fitness-contributing genes on each replicon or in each environment (Supplementary Figs 7 and 8). The robustness of these results to environmental variations provides support for the validity of our conclusions and shows that the niche specialization is not unique to the specific environmental composition used throughout this study.

Finally, a comparison of genes differentially contributing to growth in each environment with a recent regulon analysis in *S. meliloti*^12^ was not conclusive due to the low overlap of the data sets (Supplementary Table 4 and Supplementary Data 5; additional details in Supplementary Note 4). On the other hand, grouping these genes based on their pangenome classification^12^ illustrated that nearly all fitness-contributing genes belonged to the core genome, a clear enrichment relative to the percentage of core genes in iGD1575 overall (Supplementary Table 5 and Supplementary Data 5; additional details in Supplementary Note 5).

## Discussion

We have completed a comprehensive, manually and experimentally curated genome-scale metabolic reconstruction of a model multipartite genome of the N_2_-fixing endosymbiont *S. meliloti*, and modelled the metabolic changes associated with niche transition. The switch from bulk soil to the rhizosphere was accompanied by a metabolic fine-tuning, primarily through changes in carbon metabolism and amino-acid biosynthesis. In contrast, moving from the rhizosphere to the nodule involved a comprehensive metabolic reprogramming. This involved essentially shutting off production of all biomass compounds and instead synthesizing co-factors necessary for a successful symbiosis, maximizing ATP production and fixing atmospheric nitrogen.

The analysis of the *in silico* fitness contributions of genes included in iGD1575 revealed that the chromosome is not metabolically specialized for a particular niche, but instead encodes the core metabolic machinery that enables growth of *S. meliloti* as a free-living microbe. In contrast, the evidence indicated that pSymB is metabolically specialized for the rhizosphere, helping *S. meliloti* to adapt to this environment and utilize the newly available substrates. The analysis failed to detect any environment where pSymA contributed to improved fitness, but it was seen that pSymA functions were solely relevant to the symbiotic process. Concerning multipartite genome evolution, these observations are consistent with an evolutionary scenario where (1) the gain of pSymB first significantly improved the ability of *S. meliloti* to colonize the rhizosphere as suggested previously^4^,^55^, (2) pSymB gained additional genes encoding metabolic functions that contribute to fitness predominately in the rhizosphere and (3) pSymA only contributes metabolic functions relevant for establishing a N_2_-fixing symbiosis.

We speculate that our observations here with *S. meliloti* may be generalizable to other bacteria with a multipartite genome that interact with a eukaryotic host. We hypothesize that secondary replicons might facilitate the start of a host interaction; this is the case for the large *Escherichia coli* virulence plasmids^7^ and the rhizobial symbiotic plasmids^18^. Once the organism begins inhabiting the host-associated niche, the secondary replicon might acquire genes that improve fitness specifically in this new environment, whereas the chromosome remains largely undifferentiated, carrying the general metabolic pathways required for life and traits specific to the cell's original environment.

The modelling framework we have developed for this work can be adapted to study other types of biological association (for example, pathogenesis) and the metabolic reprogramming that is needed to operate the switch towards a novel ecological niche. Moreover, by demonstrating here that chromids and megaplasmids carry genes that primarily improve fitness in a specific niche, such as host interaction, this work illustrates secondary replicons as a rich reservoir of genes that have potential in synthetic biology applications. Finally, we anticipate that the iGD1575 model herein reconstructed will represent a valuable platform for future manipulations of *S. meliloti* aimed at its biotechnological exploitation in the context of agricultural procedures.

## Methods

### Metabolic network reconstruction

A draft metabolic model was constructed using the KBase Narrative Interface (www.kbase.us) and then manually and experimentally validated and expanded based on published data as described in the Supplementary Methods. The final *S. meliloti* model was termed iGD1575 in accordance with the nomenclature standard^56^, and includes 1,575 genes, 1,825 reactions and 1,579 metabolites. The SBML file of the model was validated by the online SBML validator tool (http://sbml.org/Facilities/Validator/), and is available as Supplementary Data 6. Metabolic modelling was performed using Matlab R2015a (Mathworks), using scripts from the Cobra Toolbox^57^ and the Gurobi 6.0.1 solver (www.gurobi.com). A detailed description of the modelling procedure is reported in the Supplementary Methods. For comparison of iGD1575 with previously published flux data^23^, the flux through each reaction was constrained by setting the upper and lower bounds to the average plus or minus the error of the experimentally derived values. To facilitate construction of the *in silico* large-scale *S. meliloti* gene deletion mutants in iGD1575, identification of essential model genes was performed as described in Supplementary Note 6 and the essential iGD1575 genes are listed in Supplementary Table 7.

### Biomass composition

No comprehensive description of the macromolecular composition of the *S. meliloti* biomass exists in the literature. However, such data are available for *Rhodobacter sphaeroides*, a related α-proteobacterium^58^. We therefore approximated the *S. meliloti* gross biomass composition using that of *R. sphaeroides*. Nevertheless, the specific composition of DNA, RNA, protein and lipids was adjusted based on the *S. meliloti* GC content^13^, codon usage^59^ and lipid composition^60^,^61^,^62^,^63^. Furthermore, succinoglycan was included in the biomass at 5% of the dry weight, which was estimated based on the literature^64^,^65^,^66^. The complete biomass composition is given in Supplementary Table 2.

### Objective function formulation

The objective function for growth in synthetic media and bulk soil was set as a biomass reaction, producing biomass as described in the above section and fully detailed in Supplementary Table 2; this objective function was termed ‘biomass_bulk_c0'. The objective function for growth in the rhizosphere (biomass_rhizo_c0) was the same as for bulk soil except that the amount of succinoglycan was doubled to account for biofilm formation on the plant root, and Nod factor was included (1 mg per g dry weight) as its production would be stimulated by the legume and is required for the initiation of symbiosis (Supplementary Table 2). Finally, the ‘symbiosis_c0' objective function was adapted from a published *S. meliloti* model^19^, and was used for modelling symbiosis. In short, the symbiosis objective function involved the synthesis of biomolecules relevant to symbiosis, as well as the export of L-alanine, L-aspartate and ammonium from fixed N_2_.

### *In silico* environmental representations

*In silico* representations of the nutritional composition of the rhizosphere and bulk soil were constructed from data available in the literature (Table 3). For both soil representations, ammonium and nitrate were included at a one to one ratio, and sufficient ammonium, nitrate, phosphate, sulphate, metal ions and gases were included so that these compounds were not growth rate limiting. The relative abundance of the major carbon compounds was derived from the available literature as described in the Supplementary Methods. The boundaries of the exchange reactions used to define each environment are listed in Supplementary Table 6, as are the flux rate through all active exchange reactions.

### Gene functional analysis

The WebMGA webserver^67^ was used to provide functional COG annotations (*P* value cutoff of 0.001) to each gene in the model. Between-replicon biases were determined after standardizing by the number of genes from each replicon in iGD1575. To perform the COG analyses of the genes associated with variable reactions during the transition between niches, the COG annotation for each gene associated with the variable reaction classes was extracted from the WebMGA output of the previous COG analysis. Biases were determined after standardizing by the number of genes in each class of variable genes. Statistical significance was determined using Pearson's *χ*^2^-tests. The complete list of COG annotations is available as Supplementary Data 7.

### Phenotype MicroArray analysis

Phenotype MicroArray experiments using Biolog plates PM1 and PM2A were performed largely as described previously^25^,^49^ with details elaborated on in the Supplementary Methods. All bacterial strains used in this study were described previously^31^,^49^ and are listed in Supplementary Table 8. Of note, whereas most strains were inoculated from agar plates, *S. meliloti* RmP2754 (ΔB180) and a second wild-type control were inoculated from liquid M9-glucose cultures as RmP2754 grew poorly when inoculated directly from an agar plate. Data analysis was performed with DuctApe^27^. Activity index (AV) values were calculated following subtraction of the blank well from the experimental wells, whereas plots of the growth curves are of the unblanked data. Growth with each compound was considered positive if the AV value was ≥4. Negative growth phenotypes of the mutant strains were called if the AV value was ≤3, and only following manual inspection of the unblanked curves. However, it must be noted that a growth cutoff of 4 is likely to falsely eliminate some compounds that support slow growth of *S. meliloti*^27^, such as beta-hydroxybutyrate (AV value=3) and acetoacetate (AV value=2)^68^.

### Growth curves and phosphate determination

*S. meliloti* was grown overnight in LBmc complex medium^4^. These cultures were washed with 0.85% saline and resuspended to an OD_600_ of ∼0.05 in MM9 minimal medium^4^ with either 7.5 mM glucose or 20 mM succinate as the sole carbon source. A volume of 200 μl of the cell suspensions were transferred in triplicate to wells of a 96-well microtitre plates and grown for 24 h at 30 °C with shaking in a BioTek Cytation 3 plate reader. OD_600_ readings were measured every 15 min. Growth rates were calculated between OD_600_ readings (not corrected for pathlength) of 0.1–0.5 with a previously developed Perl script^4^.

To measure the amount of phosphate used by *S. meliloti*, the phosphate concentrations in the spent media following the completion of the growth curves were determined via the molybdenate blue—ascorbic acid colorimetric method^69^. In brief, cultures were centrifuged, 50 μl of supernatant was diluted with 5 ml of phosphate-free water and 0.8 ml of mixed reagent^69^ was added to each sample. Following 10–30 min of incubation at room temperature, the A_880_ of each sample was measured and compared with a standard curve. The amount of phosphate remaining in spent media was compared with the phosphate present in the bacteria-free cultures to determine the amount of phosphate used by the bacteria. As the carbon source is growth limiting in these media, the used phosphate to carbon source ratio was calculated by dividing the amount of phosphate removed from the medium by the initial concentration of the carbon source (that is, 7.5 mM glucose or 20 mM succinate).

### Data availability

The authors declare that the data supporting the findings of this study are available within the article and its Supplementary Information files. Matlab scripts used for generation of the FBA data are available from the authors on request.

## Additional information

**How to cite this article:** diCenzo, G. C. *et al*. Metabolic modelling reveals the specialization of secondary replicons for niche adaptation in *Sinorhizobium meliloti*. *Nat. Commun.* 7:12219 doi: 10.1038/ncomms12219 (2016).

## Supplementary Material

Supplementary Figures, Supplementary Tables, Supplementary Notes, Supplementary Methods and Supplementary References Supplementary Figures 1-8, Supplementary Tables 1-8, Supplementary Notes 1-6, Supplementary Methods and Supplementary References

Supplementary Data 1 Contains the analyzed data of all Phenotype MicroArrayTM data (experimental and in silico) used or generated in this study.

Supplementary Data 2 Contains all the raw Phenotype MicroArrayTM data that was generated in this study in the form of .csv files.

Supplementary Data 3 Contains tables that list all the reactions showing different characteristics during growth in bulk soil versus the rhizosphere.

Supplementary Data 4 Contains tables that list all the reactions showing different characteristics during growth in the rhizosphere versus the nodule.

Supplementary Data 5 Contains previously published pangenome information and regulon data for all genes that are included in iGD1575.

Supplementary Data 6 The sbml file of iGD1575.

Supplementary Data 7 Contains the COG annotations for all genes included within iGD1575, as generated by WebMGA.

## Figures and Tables

**Table 1:** Summary of the main properties of iGD1575.

S. meliloti *1021 genome*	
Total genome size	6,691,694
Size of the chromosome (% total)	3,654,135 (54.6)
Size of pSymB (% total)	1,683,333 (25.2)
Size of pSymA (% total)	1,354,226 (20.2)
Total protein-coding genes (PCG)	6 204
Chromosome PCG (% total)	3,341 (53.9)
pSymB PCG (% total)	1,570 (25.3)
pSymA PCG (% total)	1,293 (20.8)
	
*iGD1575 characteristics*	
Total genes (% of *S. meliloti* genes)	1,575 (25.4)
Chromosome genes (% total)	944 (69.9)
pSymB genes (% total)	390 (24.8)
pSymA genes (% total)	241 (15.3)
Total reactions (rxns)	1,825
Gene-associated rxns (gar) (% total)	1,404 (76.9)
Chromosome dependent (% gar)	898 (64.0)
pSymB dependent (% gar)	205 (14.6)
pSymA dependent (% gar)	73 (5.2)
Multiple replicons (% gar)	228 (16.2)
Unknown metabolic GPR (% total)	63 (3.4)
Unknown transport GPR (% total)	46 (2.5)
Exchange reactions (% total)*	270 (14.8)
Demand reactions (% total)†	22 (1.2)
Diffusion reactions (% total)	8 (0.4)
Spontaneous reactions (% total)	10 (0.5)
Objective functions (% total)	3 (0.2)
Total metabolites	1,579

GPR, Gene-Protein-Reaction.

^*^Exchange reactions are used to define the medium components.

^†^Demand reactions are used to provide compounds whose synthesis is not represented in the model. In all, 20 of the demand reactions represent the uncharged tRNA molecules, 2 are for fatty acids.

**Table 2:** Carbon utilization phenotypes observed for pSymB deletion mutants.

Strain	Phenotypes		
	Biolog	Both	Model
ΔB154	L-histidine	None	None
	D-glucosamine		
ΔB141	Palatinose*	D-trehalose	*Cis*-4-hydroxy-D-proline†
	Maltitol‡	*Trans*-4-hydroxy-L-proline	
ΔB163	Dulcitol*	D-psicose	None
	D-glucosamine		
ΔB180	Uridine*	L-leucine	D-galactosamine§
	*N*-acetyl-D-galactosamine*		
	Arbutin*		
	D-raffinose		
	Organic acids||		
ΔB181	L-lysine*	L-histidine	D-galactosamine§
	Dulcitol*	D-tagatose	
	*N*-acetyl-D-galactosamine*		
	D-glucosamine		
ΔB108	L-ornithine	None	None
	L-serine		
	L-asparagine		
	L-alanine		
ΔB109	None	None	None
ΔB179	Glycerol	L-arabinose	None
	L-lactic acid	M-inositol	
ΔB118	None	L-ornithine	None
		M-inositol	
		L-arginine	
ΔB182	Acetic acid	D-melibiose	Protocatechuate†
	Asparagine‡	L-malic acid	
	α-methyl-D-galactoside‡	D-raffinoseSuccinic acid	
	Melibionic acid‡	D,L-malate	
	Bromosuccinate‡	Fumaric acidL-aspartate	
ΔB161	Dulcitol*	D-melibiose	None
	α-methyl-D-galactoside‡	D-raffinoseα-D-lactose	
	Melibionic acid‡	LactuloseMethyl-β-D-galactoside	

The ‘Biolog' column indicates the phenotypes observed experimentally that are not seen *in silico* with the iGD1575 model, and *vice versa* for the ‘Model' column. The ‘Both' column lists the phenotypes observed both experimentally and *in silico*.

^*^The model does not produce biomass with this compound.

^†^This compound is not present in the PM1 or PM2A plates, but the phenotype is confirmed in the literature.

^‡^The model does not include this compound.

^§^This compound is not present in the PM1 or PM2A plates, and the phenotype has not been reported in the literature.

^||^Includes all tested L-amino acids, gly–glu, ala–gly, gly–asp, L-lactic acid, acetic acid, pyruvic acid, methylpyruvic acid, melibionic acid and gamma-aminobutyric acid (GABA).

**Table 3:** Nutritional composition of the rhizosphere and bulk soil.

Compound	Rhizosphere*	Bulk soil*
Total sugars	0.615	1.753
Arabinose	0.201	0.273
Fucose†	0.030	0.064
Galactose	0.136	0.239
Galacturonic acid‡	0.053	—
Glucose	0.030	0.586
Glucuronic acid‡	0.023	—
Mannose	0.040	0.240
Rhamnose	0.022	0.132
Xylose	0.034	0.201
Ribose	—	0.018
Sucrose	0.015	0.000
Raffinose	0.008	0.000
Stachyose	0.023	0.000
Total organic acids	1.231	0.072
Succinate	0.091	0.005
Malate	0.591	0.035
Citrate‡	0.549	0.032
Total amino acids	0.154	0.175
Aspartic acid	0.024	0.000
Threonine	0.007	0.006
Serine	0.007	0.000
Homoserine‡	0.046	—
Glutamic acid	0.021	0.000
Proline	0.000	0.010
Glycine	0.005	0.006
Alanine	0.006	0.026
Valine	0.003	0.028
Cysteine	0.000	0.006
Isoleucine	0.001	0.019
Leucine	0.002	0.034
Tyrosine	0.002	0.006
Phenylalanine‡	0.002	0.006
GABA	0.000	—
Ornithine	0.006	0.006
Lysine†	0.004	0.006
Histidine	0.004	0.006
Arginine	0.007	0.006
Asparagine	—	0.000
Glutamine	—	0.000
Hydroxyproline	0.006	—
Ammonium	Excess	Excess
Nitrate	Excess	Excess
Sulfate	Excess	Excess
Phosphate	Excess	Excess

GABA, gamma-aminobutyric acid.

^*^Values represent the molar ratio of the compounds, with the sum of all compounds in each environment equalling 2. —, no information.

^†^Excluded from the *in silico* representation as the model fails to grow on these compounds.

^‡^Excluded from the *in silico* representation as *S. meliloti* does not grow with these compounds.

## References

[b1] Harrison P. W., Lower R. P. J., Kim N. K. D. & Young J. P. W. Introducing the bacterial ‘chromid': not a chromosome, not a plasmid. Trends Microbiol. 18, 141–148 (2010). 20080407 10.1016/j.tim.2009.12.010

[b2] Landeta C. . Plasmids with a chromosome-like role in rhizobia. J. Bacteriol. 193, 1317–1326 (2011). 21217003 10.1128/JB.01184-10 PMC3067620

[b3] Galardini M., Pini F., Bazzicalupo M., Biondi E. G. & Mengoni A. Replicon-dependent bacterial genome evolution: the case of *Sinorhizobium meliloti*. Genome Biol. Evol. 5, 542–558 (2013). 23431003 10.1093/gbe/evt027 PMC3622305

[b4] diCenzo G. C., MacLean A. M., Milunovic B., Golding G. B. & Finan T. M. Examination of prokaryotic multipartite genome evolution through experimental genome reduction. PLoS Genet. 10, e1004742 (2014). 25340565 10.1371/journal.pgen.1004742 PMC4207669

[b5] Rocha E. P. C. The organization of the bacterial genome. Annu. Rev. Genet. 42, 211–233 (2008). 18605898 10.1146/annurev.genet.42.110807.091653

[b6] diCenzo G. C., Zamani M., Milunovic B. & Finan T. M. Genomic resources for identification of the minimal N2-fixing symbiotic genome. Environ. Microbiol. doi:10.1111/1462-2920.13221 (2016). 26768651

[b7] Johnson T. J. & Nolan L. K. Pathogenomics of the virulence plasmids of *Escherichia coli*. Microbiol. Mol. Biol. Rev. 73, 750–774 (2009). 19946140 10.1128/MMBR.00015-09 PMC2786578

[b8] Morton E. R., Merritt P. M., Bever J. D. & Fuqua C. Large deletions in the pAtC58 megaplasmid of *Agrobacterium tumefaciens* can confer reduced carriage cost and increased expression of virulence genes. Genome Biol. Evol. 5, 1353–1364 (2013). 23783172 10.1093/gbe/evt095 PMC3730347

[b9] Romanchuk A. . Bigger is not always better: transmission and fitness burden of ∼1MB *Pseudomonas syringae* megaplasmid pMPPla107. Plasmid 73, 16–25 (2014). 24792221 10.1016/j.plasmid.2014.04.002

[b10] Xu Q., Dziejman M. & Mekalanos J. J. Determination of the transcriptome of *Vibrio cholerae* during intraintestinal growth and midexponential phase *in vitro*. Proc. Natl Acad. Sci. USA 100, 1286–1291 (2003). 12552086 10.1073/pnas.0337479100 PMC298765

[b11] Becker A. . Global changes in gene expression in *Sinorhizobium meliloti* 1021 under microoxic and symbiotic conditions. Mol. Plant Microbe Interact. 17, 292–303 (2004). 15000396 10.1094/MPMI.2004.17.3.292

[b12] Galardini M. . Evolution of intra-specific regulatory networks in a multipartite bacterial genome. PLoS Comput. Biol. 11, e1004478 (2015). 26340565 10.1371/journal.pcbi.1004478 PMC4560400

[b13] Galibert F. . The composite genome of the legume symbiont *Sinorhizobium meliloti*. Science 293, 668–672 (2001). 11474104 10.1126/science.1060966

[b14] Galardini M. . Exploring the symbiotic pangenome of the nitrogen-fixing bacterium *Sinorhizobium meliloti*. BMC Genomics 12, 235 (2011). 21569405 10.1186/1471-2164-12-235 PMC3164228

[b15] Hinsinger P., Bengough A. G., Vetterlein D. & Young I. M. Rhizosphere: biophysics, biogeochemistry and ecological relevance. Plant Soil 321, 117–152 (2009).

[b16] Oldroyd G. E. & Dixon R. Biotechnological solutions to the nitrogen problem. Curr. Opin. Biotechnol. 26, 19–24 (2014). 24679253 10.1016/j.copbio.2013.08.006

[b17] Geddes B. A. . Use of plant colonizing bacteria as chassis for transfer of N_2_-fixation to cereals. Curr. Opin. Biotechnol. 32, 216–222 (2015). 25626166 10.1016/j.copbio.2015.01.004

[b18] Remigi P., Zhu J., Young J. P. W. & Masson-Boivin C. Symbiosis within symbiosis: evolving nitrogen-fixing legume symbionts. Trends Microbiol. 24, 63–75 (2016). 26612499 10.1016/j.tim.2015.10.007

[b19] Zhao H., Li M., Fang K., Chen W. & Wang J. *In silico* insights into the symbiotic nitrogen fixation in *Sinorhizobium meliloti* via metabolic reconstruction. PLoS ONE 7, e31287 (2012). 22319621 10.1371/journal.pone.0031287 PMC3272708

[b20] Schellenberger J., Park J. O., Conrad T. M. & Palsson B. Ø. BiGG: a Biochemical Genetic and Genomic knowledgebase of large scale metabolic reconstructions. BMC Bioinformatics 11, 213 (2010). 20426874 10.1186/1471-2105-11-213 PMC2874806

[b21] Resendis-Antonio O., Reed J. L., Encarnación S., Collado-Vides J. & Palsson B. Ø. Metabolic reconstruction and modeling of nitrogen fixation in *Rhizobium etli*. PLoS Comput. Biol. 3, 1887–1895 (2007). 17922569 10.1371/journal.pcbi.0030192 PMC2000972

[b22] Resendis-Antonio O. . Systems biology of bacterial nitrogen fixation: high-throughput technology and its integrative description with constraint-based modeling. BMC Syst. Biol. 5, 120 (2011). 21801415 10.1186/1752-0509-5-120 PMC3164627

[b23] Fuhrer T., Fischer E. & Sauer U. Experimental identification and quantification of glucose metabolism in seven bacterial species. J. Bacteriol. 187, 1581–1590 (2005). 15716428 10.1128/JB.187.5.1581-1590.2005 PMC1064017

[b24] Yurgel S., Mortimer M. W., Rogers K. N. & Kahn M. L. New substrates for the dicarboxylate transport system of *Sinorhizobium meliloti*. J. Bacteriol. 182, 4216–4221 (2000). 10894729 10.1128/jb.182.15.4216-4221.2000 PMC101915

[b25] Biondi E. G. . Metabolic capacity of *Sinorhizobium* (*Ensifer*) *meliloti* strains as determined by Phenotype MicroArray analysis. Appl. Environ. Microbiol. 75, 5396–5404 (2009). 19561177 10.1128/AEM.00196-09 PMC2725449

[b26] Spini G. . Effect of the plant flavonoid luteolin on *Ensifer meliloti* 3001 phenotypic responses. Plant Soil 399, 159–178 (2015).

[b27] Galardini M. . DuctApe: a suite for the analysis and correlation of genomic and OmniLog Phenotype Microarray data. Genomics 103, 1–10 (2014). 24316132 10.1016/j.ygeno.2013.11.005

[b28] Fondi M. . Genome-scale metabolic reconstruction and constraint-based modelling of the Antarctic bacterium *Pseudoalteromonas haloplanktis* TAC125. Environ. Microbiol. 17, 751–766 (2014). 24889559 10.1111/1462-2920.12513

[b29] Bartell J. A., Yen P., Varga J. J., Goldberg J. B. & Papin J. A. Comparative metabolic systems analysis of pathogenic *Burkholderia*. J. Bacteriol. 196, 210–226 (2014). 24163337 10.1128/JB.00997-13 PMC3911241

[b30] Schatschneider S. . Establishment, *in silico* analysis, and experimental verification of a large-scale metabolic network of the xanthan producing *Xanthomonas campestris* pv. campestris strain B100. J. Biotechnol. 167, 123–134 (2013). 23395674 10.1016/j.jbiotec.2013.01.023

[b31] Milunovic B., diCenzo G. C., Morton R. A. & Finan T. M. Cell growth inhibition upon deletion of four toxin-antitoxin loci from the megaplasmids of *Sinorhizobium meliloti*. J. Bacteriol. 196, 811–824 (2014). 24317400 10.1128/JB.01104-13 PMC3911179

[b32] Cheng J., Poduska B., Morton R. A. & Finan T. M. An ABC-type cobalt transport system is essential for growth of *Sinorhizobium meliloti* at trace metal concentrations. J. Bacteriol. 193, 4405–4416 (2011). 21725018 10.1128/JB.05045-11 PMC3165532

[b33] Malhotra H. C., Prakash J. & Sharma G. C. Kinetics of chelation of Co(II) with L-histidine. Proc. Indian Natl Sci. Acad. 53, 223–231 (1986).

[b34] Lerivrey J. . Formation of D-glucosamine complexes with Cu(II), Ni(II) and Co(II) ions. Inorg. Chim. Acta 125, 187–190 (1986).

[b35] Mauchline T. H. . Mapping the *Sinorhizobium meliloti* 1021 solute-binding protein-dependent transportome. Proc. Natl Acad. Sci. USA 103, 17933–17938 (2006). 17101990 10.1073/pnas.0606673103 PMC1635973

[b36] Yamada T., Letunic I., Okuda S., Kanehisa M. & Bork P. iPath2.0: interactive pathway explorer. Nucleic Acids Res. 39, W412–W415 (2011). 21546551 10.1093/nar/gkr313 PMC3125749

[b37] Schmid J., Sieber V. & Rehm B. Bacterial exopolysaccharides: biosynthesis pathways and engineering strategies. Front. Microbiol. 6, 496 (2015). 26074894 10.3389/fmicb.2015.00496 PMC4443731

[b38] Carlson R. W., Price N. P. & Stacey G. The biosynthesis of rhizobial lipo-oligosaccharide nodulation signal molecules. Mol. Plant Microbe Interact. 7, 684–695 (1994). 7873777 10.1094/mpmi-7-0684

[b39] Capela D., Filipe C., Bobik C., Batut J. & Bruand C. *Sinorhizobium meliloti* differentiation during symbiosis with alfalfa: a transcriptomic dissection. Mol. Plant Microbe Interact. 19, 363–372 (2006). 16610739 10.1094/MPMI-19-0363

[b40] Barnett M. J., Toman C. J., Fisher R. F. & Long S. R. A dual-genome Symbiosis Chip for coordinate study of signal exchange and development in a prokaryote–host interaction. Proc. Natl Acad. Sci. USA 101, 16636–16641 (2004). 15542588 10.1073/pnas.0407269101 PMC527922

[b41] Djordjevic M. A. *Sinorhizobium meliloti* metabolism in the root nodule: a proteomic perspective. Proteomics 4, 1859–1872 (2004). 15221743 10.1002/pmic.200300802

[b42] Roux B. . An integrated analysis of plant and bacterial gene expression in symbiotic root nodules using laser-capture microdissection coupled to RNA sequencing. Plant J. 77, 817–837 (2014). 24483147 10.1111/tpj.12442

[b43] Prell J., Boesten B., Poole P. & Priefer U. B. The *Rhizobium leguminosarum* bv. *viciae* VF39 γ-aminobutyrate (GABA) aminotransferase gene (*gabT*) is induced by GABA and highly expressed in bacteroids. Microbiology 148, 615–623 (2002). 11832524 10.1099/00221287-148-2-615

[b44] Geddes B. A. & Oresnik I. J. Physiology, genetics, and biochemistry of carbon metabolism in the alphaproteobacterium *Sinorhizobium meliloti*. Can. J. Microbiol. 60, 491–507 (2014). 25093748 10.1139/cjm-2014-0306

[b45] Udvardi M. & Poole P. S. Transport and metabolism in legume-rhizobia symbioses. Annu. Rev. Plant Biol. 64, 781–805 (2013). 23451778 10.1146/annurev-arplant-050312-120235

[b46] diCenzo G. C. & Finan T. M. Genetic redundancy is prevalent within the 6.7 Mb *Sinorhizobium meliloti* genome. Mol. Genet. Genomics 290, 1345–1356 (2015). 25638282 10.1007/s00438-015-0998-6

[b47] diCenzo G. C., Zamani M., Cowie A. & Finan T. M. Proline auxotrophy in *Sinorhizobium meliloti* results in a plant-specific symbiotic phenotype. Microbiology 161, 2341–2351 (2015). 26395514 10.1099/mic.0.000182

[b48] Poysti N. J., Loewen E. D. M., Wang Z. & Oresnik I. J. *Sinorhizobium meliloti* pSymB carries genes necessary for arabinose transport and catabolism. Microbiology 153, 727–736 (2007). 17322193 10.1099/mic.0.29148-0

[b49] diCenzo G., Milunovic B., Cheng J. & Finan T. M. The tRNAarg gene and *engA* are essential genes on the 1.7-mb pSymB megaplasmid of *Sinorhizobium meliloti* and were translocated together from the chromosome in an ancestral strain. J. Bacteriol. 195, 202–212 (2013). 23123907 10.1128/JB.01758-12 PMC3553834

[b50] Ramachandran V. K., East A. K., Karunakaran R., Downie J. A. & Poole P. S. Adaptation of *Rhizobium leguminosarum* to pea, alfalfa and sugar beet rhizospheres investigated by comparative transcriptomics. Genome Biol. 12, R106 (2011). 22018401 10.1186/gb-2011-12-10-r106 PMC3333776

[b51] Garcia-Fraile P. . Arabinose and protocatechuate catabolism genes are important for growth of *Rhizobium leguminosarum* biovar *viciae* in the pea rhizosphere. Plant Soil 390, 251–264 (2015). 26166901 10.1007/s11104-015-2389-5 PMC4495286

[b52] MacLean A. M., MacPherson G., Aneja P. & Finan T. M. Characterization of the β-ketoadipate pathway in *Sinorhizobium meliloti*. Appl. Environ. Microbiol. 72, 5403–5413 (2006). 16885292 10.1128/AEM.00580-06 PMC1538742

[b53] MacLean A. M., Haerty W., Golding G. B. & Finan T. M. The LysR-type PcaQ protein regulates expression of a protocatechuate-inducible ABC-type transport system in *Sinorhizobium meliloti*. Microbiology 157, 2522–2533 (2011). 21700663 10.1099/mic.0.050542-0

[b54] Yurgel S. N., Mortimer M. W., Rice J. T., Humann J. L. & Kahn M. L. Directed construction and analysis of a *Sinorhizobium meliloti* pSymA deletion mutant library. Appl. Environ. Microbiol. 79, 2081–2087 (2013). 23335760 10.1128/AEM.02974-12 PMC3592244

[b55] Egan E. S., Fogel M. A. & Waldor M. K. Divided genomes: negotiating the cell cycle in prokaryotes with multiple chromosomes. Mol. Microbiol. 56, 1129–1138 (2005). 15882408 10.1111/j.1365-2958.2005.04622.x

[b56] Reed J. L., Vo T. D., Schilling C. H. & Palsson B. Ø. An expanded genome-scale model of *Escherichia coli* K-12 (iJR904 GSM/GPR). Genome Biol. 4, R54 (2003). 12952533 10.1186/gb-2003-4-9-r54 PMC193654

[b57] Schellenberger J. . Quantitative prediction of cellular metabolism with constraint-based models: the COBRA Toolbox v2.0. Nat. Protoc. 6, 1290–1307 (2011). 21886097 10.1038/nprot.2011.308 PMC3319681

[b58] Imam S. . iRsp1095: a genome-scale reconstruction of the *Rhodobacter sphaeroides* metabolic network. BMC Syst. Biol. 5, 116 (2011). 21777427 10.1186/1752-0509-5-116 PMC3152904

[b59] Nakamura Y., Gojobori T. & Ikemura T. Codon usage tabulated from international DNA sequence databases: status for the year 2000. Nucleic Acids Res. 28, 292 (1999). 10.1093/nar/28.1.292 PMC102460 10592250

[b60] Weissenmayer B., Gao J. L., López-Lara I. M. & Geiger O. Identification of a gene required for the biosynthesis of ornithine-derived lipids. Mol. Microbiol. 45, 721–733 (2002). 12139618 10.1046/j.1365-2958.2002.03043.x

[b61] Gao J.-L. . Identification of a gene required for the formation of lyso-ornithine lipid, an intermediate in the biosynthesis of ornithine-containing lipids. Mol. Microbiol. 53, 1757–1770 (2004). 15341653 10.1111/j.1365-2958.2004.04240.x

[b62] Zavaleta-Pastor M. . *Sinorhizobium meliloti* phospholipase C required for lipid remodeling during phosphorus limitation. Proc. Natl Acad. Sci. USA 107, 302–307 (2010). 20018679 10.1073/pnas.0912930107 PMC2806695

[b63] Basconcillo L. S., Zaheer R., Finan T. M. & McCarry B. E. A shotgun lipidomics study of a putative lysophosphatidic acid acyl transferase (PlsC) in *Sinorhizobium meliloti*. J. Chromatogr. B 877, 2873–2882 (2009). 10.1016/j.jchromb.2009.05.014 19525157

[b64] Wang C. . Roles of poly-3-hydroxybutyrate (PHB) and glycogen in symbiosis of *Sinorhizobium meliloti* with *Medicago* sp. Microbiology 153, 388–398 (2007). 17259610 10.1099/mic.0.29214-0

[b65] Dorken G., Ferguson G. P., French C. E. & Poon W. C. K. Aggregation by depletion attraction in cultures of bacteria producing exopolysaccharide. J. R. Soc. Interface 9, 3490–3502 (2012). 22896568 10.1098/rsif.2012.0498 PMC3481587

[b66] Glenn S. A., Gurich N., Feeney M. A. & González J. E. The ExpR/Sin quorum-sensing system controls succinoglycan production in *Sinorhizobium meliloti*. J. Bacteriol. 189, 7077–7088 (2007). 17644606 10.1128/JB.00906-07 PMC2045190

[b67] Wu S., Zhu Z., Fu L., Niu B. & Li W. WebMGA: a customizable web server for fast metagenomic sequence analysis. BMC Genomics 12, 444 (2011). 21899761 10.1186/1471-2164-12-444 PMC3180703

[b68] Charles T. C., Cai G. Q. & Aneja P. Megaplasmid and chromosomal loci for the PHB degradation pathway in *Rhizobium (Sinorhizobium) meliloti*. Genetics 146, 1211–1220 (1997). 9258668 10.1093/genetics/146.4.1211 PMC1208069

[b69] Murphy J. & Riley J. P. A modified single solution method for the determination of phosphate in natural waters. Anal. Chim. Acta 27, 31–36 (1962).

