# Supplementary material for: Metabolic modelling reveals the specialization of secondary replicons for niche adaptation in Sinorhizobium meliloti
Source: Nat Commun. 2016 Jul 22;7:12219. doi: 10.1038/ncomms12219 (PMC4961836; doi:10.1038/ncomms12219)
Supplement: Supplementary Figures, Supplementary Tables, Supplementary Notes, Supplementary Methods and Supplementary References — Supplementary Figures 1-8, Supplementary Tables 1-8, Supplementary Notes 1-6, Supplementary Methods and Supplementary References [file ncomms12219-s1.pdf]

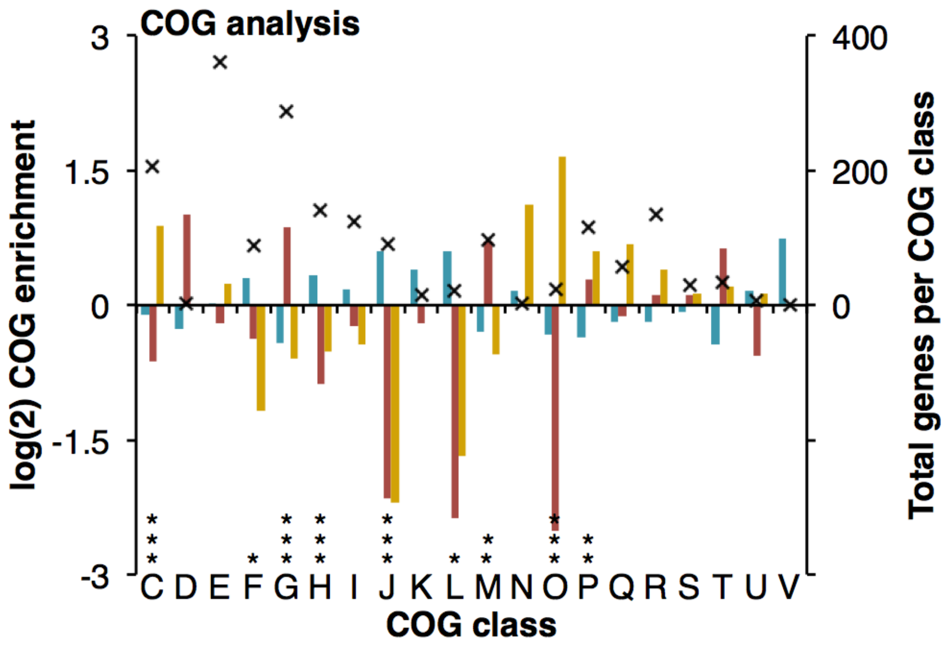

Supplementary Figure 1. COG analysis of the iGD1575 model genes. The total number of genes annotated with each COG class is represented by the crosses, which are plotted on the right axis. For each COG class represented in the gene set, the relative enrichment of each class for each replicon is shown and plotted on the left axis: blue – chromosome, red – pSymB, gold – pSymA. Statistically significant biases in distribution, as determined by Pearson's Chi Squared tests, are indicated by the asterisks: * p-value < 0.05, ** p-value < 0.01, *** p-value < 0.001. The observed biases are consistent with the previously reported COG biases for the entire *S. meliloti* chromosome, pSymA, and pSymB replicons[1,2].

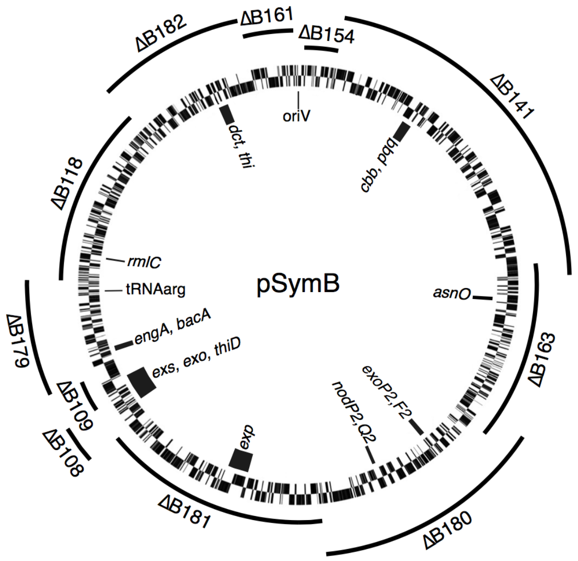

Strain	Deleted region (nucleotide position)	Strain	Deleted region (nucleotide position)
ΔB154	62,137-100,636	ΔB109	1,180,466-1,204,770
ΔB141	101,396-466,499	ΔB179	1,207,052-1,322,226
ΔB163	451,557-651,863	ΔB118	1,33,078-1,528,150
ΔB180	635,940-869,642	ΔB182	1,529,711-1,677,882
ΔB181	870,505-1,129,758	ΔB161	1,679,723-49,523
ΔB108	1,131,168-1,169,073		

Supplementary Figure 2. Deletion library mutants screened for carbon metabolic phenotypes. A schematic representation of pSymB and the location of the deletions in the studied mutants is shown. The inner circle represents pSymB, with the individual lines showing the position of annotated genes. The outer lines indicate the region of pSymB that has been removed in the corresponding deletion mutant. Several notable loci are indicated along the inner circle for reference. *dct*: *dctA,B,D*. *thi*: *thiC,O,G,E*. *exs*: *exsA-I*. *exo*: *exoA,B,F1,H,I,K-Q,T-Z*. *exp*: *wgeA-H, wgdA,B, wggR, wgcA, wgaA,B,D-J*. *cbb*: *cbbA,F,L,P,R,S,T,X*. *pqq*: *pqqA-E*.

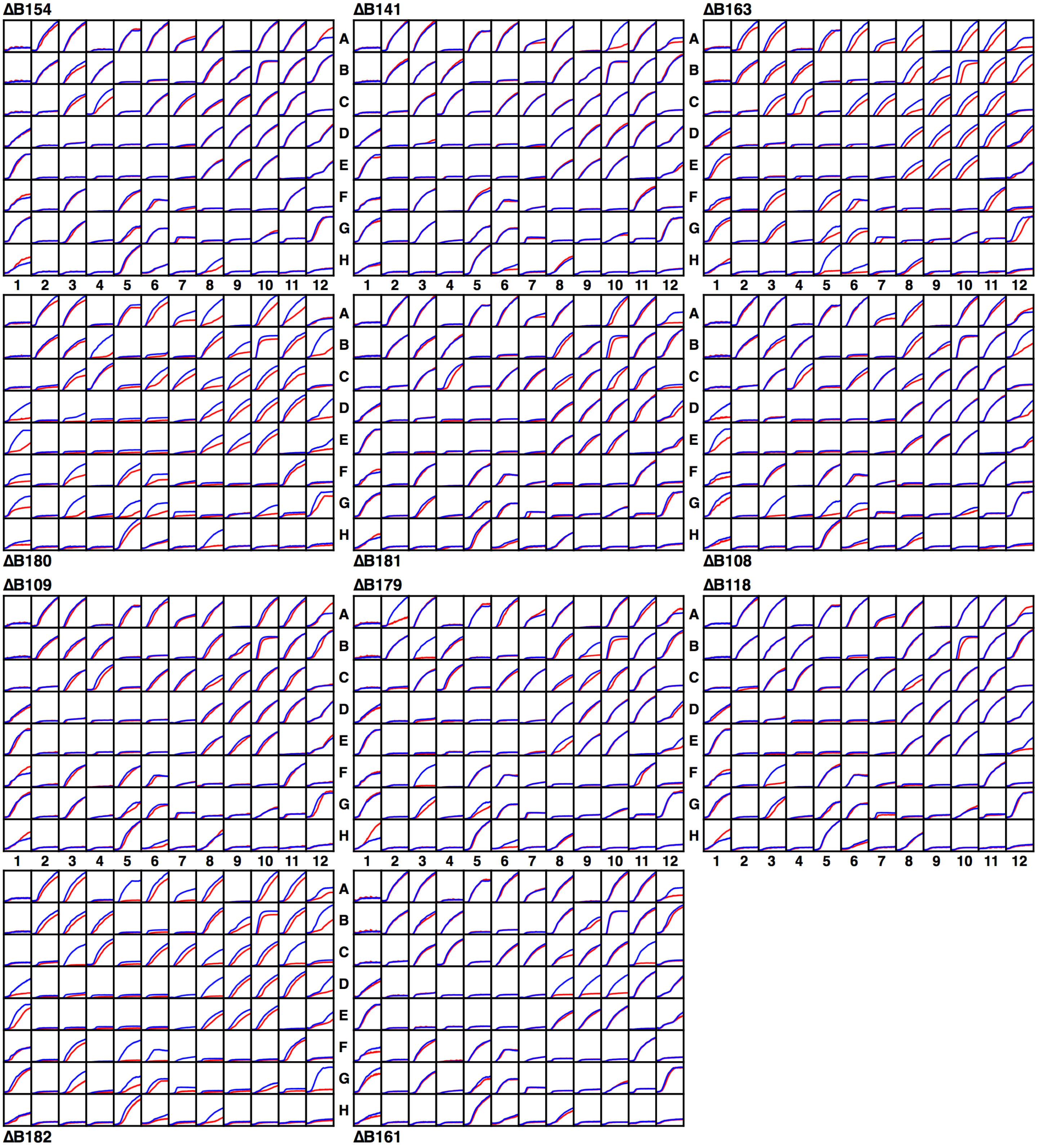

Supplementary Figure 3. Metabolic activity in the PM1 plates. Pairwise comparisons of the growth of *S. meliloti* RmP110 (blue) and the indicated deletion mutant (red) in each well of the PM1 plates. Wells are not blanked with the carbon free well (A1).

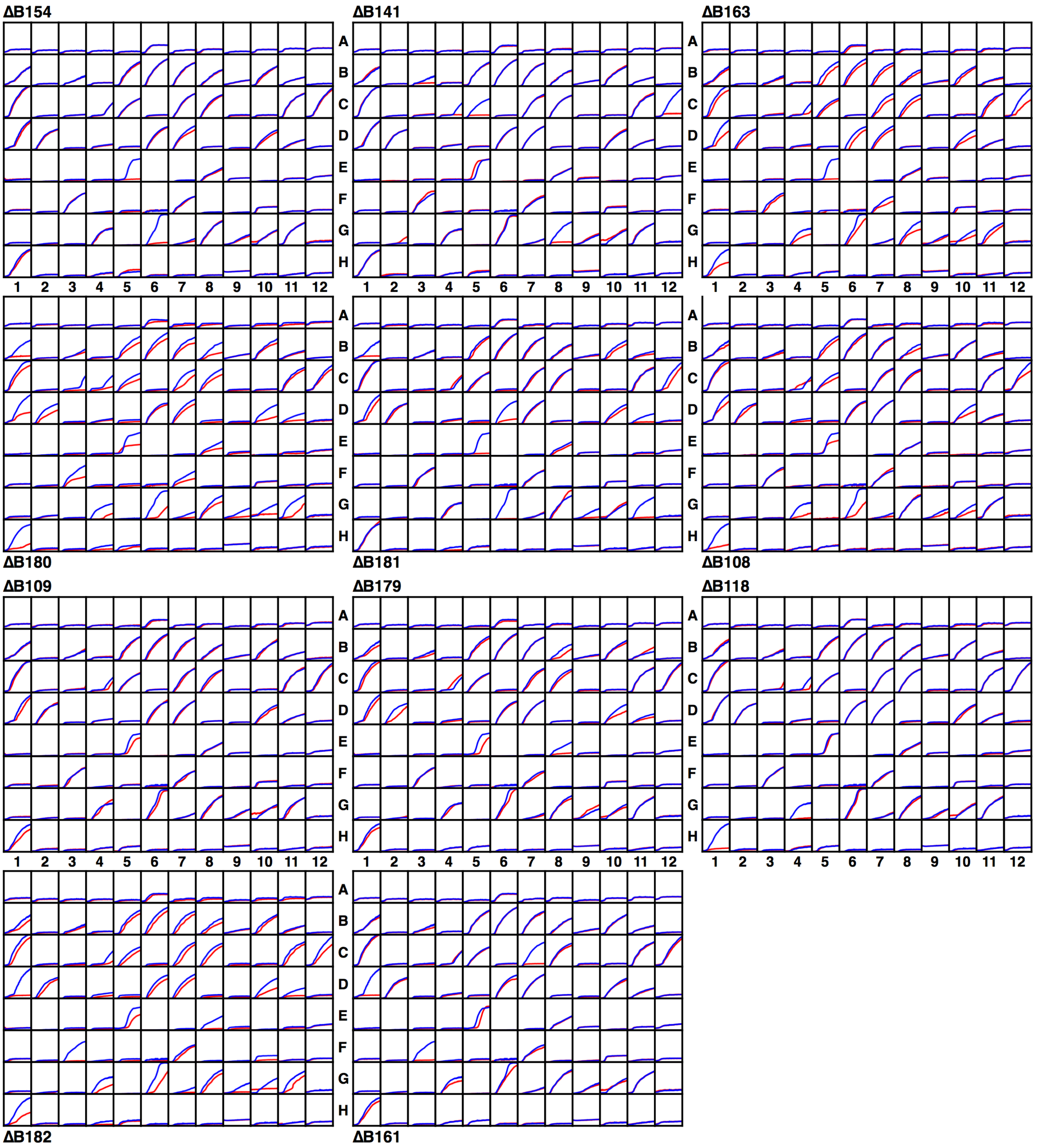

Supplementary Figure 4. Metabolic activity in the PM2A plates. Pairwise comparisons of the growth of *S. meliloti* RmP110 (blue) and the indicated deletion mutant (red) in each well of the PM2A plates. Wells are not blanked with the carbon free well (A1).

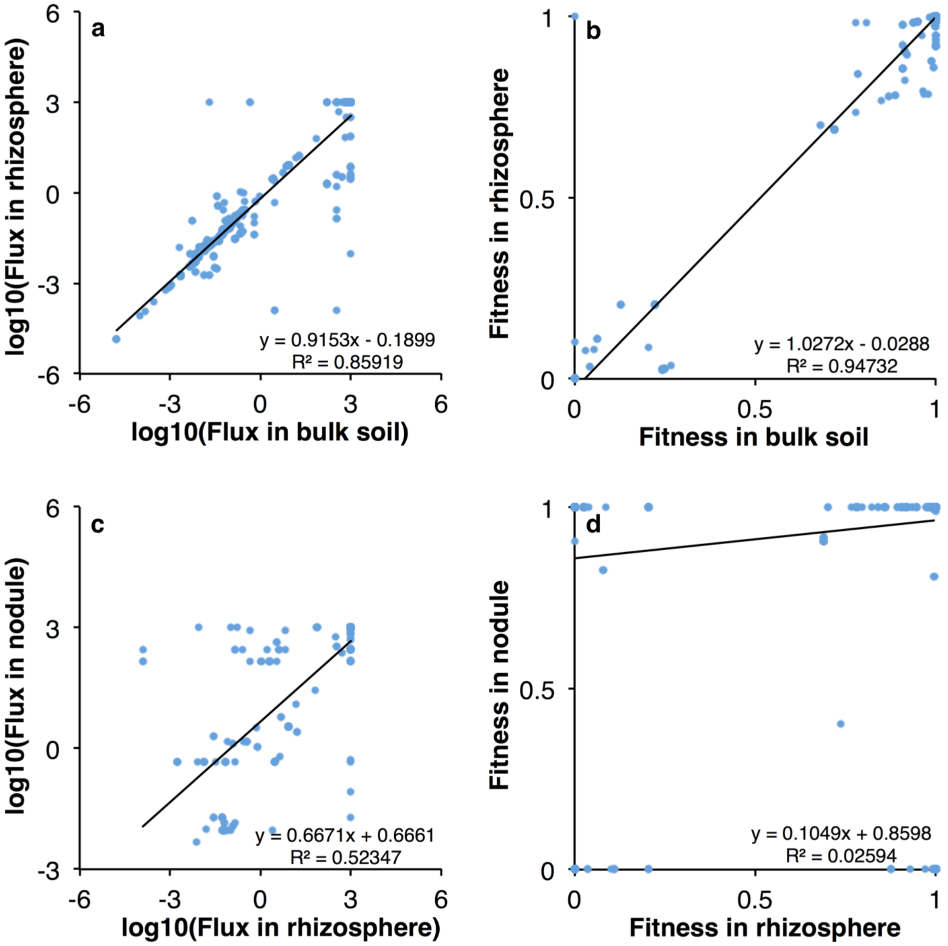

Supplementary Figure 5. Changes in reaction flux/essentiality during environmental transitions. Scatterplots, linear regression lines, equations, and R^2 values (all determined in Microsoft Excel) are shown for (**a**) individual reaction flux in bulk soil and the rhizosphere (**b**) fitness of individual reaction deletion mutants in bulk soil and the rhizosphere (**c**) individual reaction flux in the rhizosphere and the nodule (**d**) fitness of individual reaction deletion mutants in the rhizosphere and the nodule.

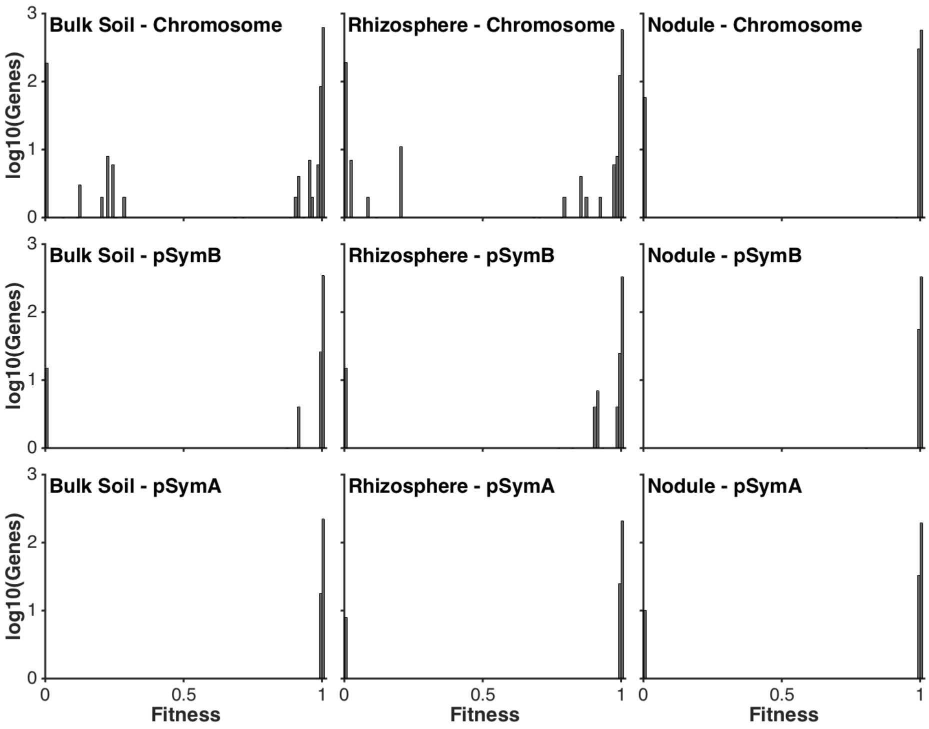

Supplementary Figure 6. Fitness costs associated with single gene deletions during growth in bulk soil, the rhizosphere, and the nodule. All genes present within iGD1575 were individually removed from the model, the ability of the resulting mutants to produce flux through the objective function was examined with FBA, and the fitness (flux through objective function in the mutant / flux through the objective function in the wild type) of each mutant was calculated. The histograms summarize the calculated fitness values for each mutant in each of the three environments, plotted separately for each replicon.

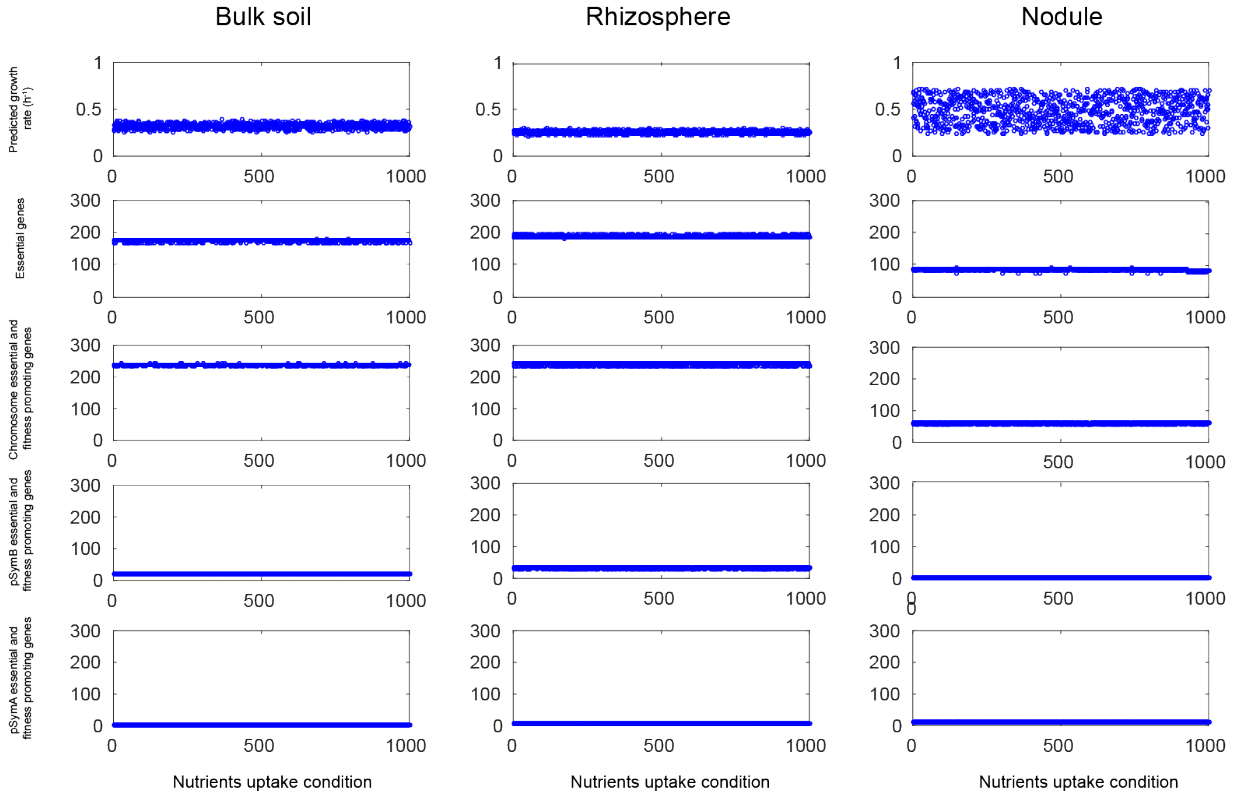

Supplementary Figure 7. Robustness to nutrients composition variation of fitness costs associated with single gene deletions and predicted growth rates. To account for the influence of the nutrients composition of the three niches analysed (bulk soil, rhizosphere, and nodule), 1000 iterations were performed and, for each iteration, new random uptake rates were generated. For each of these iterations, all genes present within iGD1575 were individually removed from the model, the ability of the resulting mutants to produce flux through the objective function was examined with FBA, and the fitness (flux through objective function in the mutant / flux through the objective function in the wild type) of each mutant was calculated. This, in turn, was repeated for each ecological niche (bulk soil, rhizosphere, and nodule). Essential and fitness promoting genes include all genes whose deletion decreases flux through the objective function by a value greater than or equal to 1%.

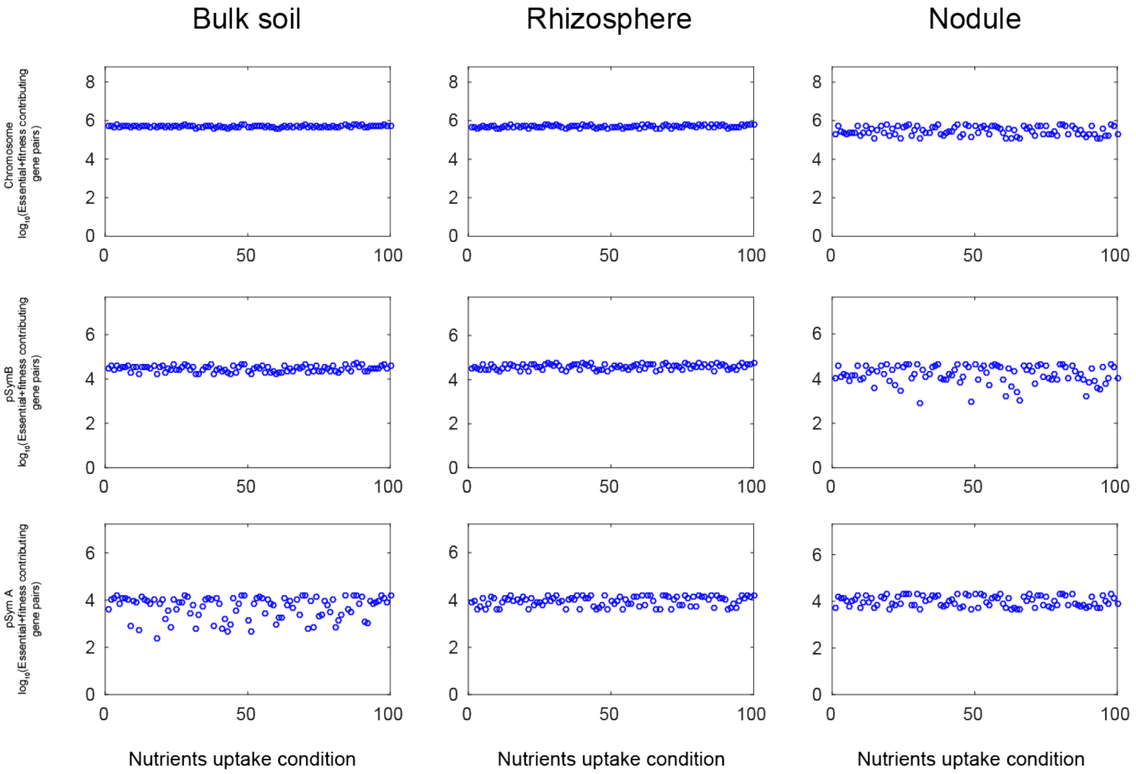

Supplementary Figure 8. Robustness to nutrients composition variation of fitness costs associated with replicon specific double gene deletions. To account for the influence of the nutrients composition of the three niches analysed (bulk soil, rhizosphere, and nodule) 100 iterations were performed and, for each iteration, new random uptake rates were generated. For each of these iterations, all the replicon specific gene pairs were removed from the model and the fitness (flux through objective function in the mutant / flux through the objective function in the wild type) of each double mutant was calculated. This, in turn, was repeated for each ecological niche (bulk soil, rhizosphere, and nodule). Essential and fitness promoting gene pairs include all gene pairs whose deletion decreases flux through the objective function by a value greater than or equal to 1%.

Supplementary Table 1. iHZ565 model genes excluded from iGD1575.

Gene	iHZ565 reaction	Reason for exclusion from iGD1575
smc04455	acetolactate synthase	Does not complement auxotrophy of a *smc01431* mutation[3]
smc03785	ADPribose diphosphatase	Required a dead end metabolite
smc03763	DNA (cytosine5)methyltransferase	Would produce a dead end metabolite
smc03243	Dihydroneopterin dephosphorylase	Too general of an annotation in the *S. meliloti* 1021 genome annotation
smc03236	GMP synthase	Lacked confidence in the annotation
smc00356	LysyltRNA synthetase	Lacked confidence in the annotation
smc02689	Aminobutyraldehyde dehydrogenase	Too general of an annotation in the *S. meliloti* 1021 genome annotation
smc02377	putrescine oxidase	Lacked confidence in the annotation
smc02263	acetolactate synthase	Does not complement auxotrophy of a *smc01431* mutation[3]
smc01404	Aspartate racemase	Would produce a dead end metabolite
smc01153	3hydroxyacylCoA dehydratase (3hydroxytetradecanoylCoA)	Too general of an annotation in the *S. meliloti* 1021 genome annotation
smc01147	coproporphyrinogen oxidase (O2 required)	Lacked confidence in the annotation
smc01146	thiamin pyrophosphatase	Lacked confidence in the annotation
smc00810	Guanosine 3'diphosphate 5'triphosphate	Lacked confidence in the annotation
smc00808	protoporphyrinogen oxidase (aerobic)	Lacked confidence in the annotation
smc00410	NADH dehydrogenase	Lacked confidence in the annotation
smb21586	glutathione synthetase	Does not complement mutation of *gshB1*[4]
smb21301	Aminobutyraldehyde dehydrogenase	Too general of an annotation in the *S. meliloti* 1021 genome annotation
smb20857	glucose6phosphate isomerase	Excluded based on [5]
smb20433	ornithine cyclodeaminase	Does not complement mutation of *ocd*[6]
smb20115	Dihydroxyacid dehydratase (2,3dihydroxy3methylpentanoate)	Does not complement auxotrophy of a *smc04045* mutation[3]
smb20072	Cytosolic source for myoinositol	Lacked confidence in the annotation
sma2213	Aminobutyraldehyde dehydrogenase	Too general of an annotation in the *S. meliloti* 1021 genome annotation
sma2211	acetolactate synthase	Does not complement auxotrophy of a *smc01431* mutation[3]
sma1871	ornithine cyclodeaminase	Does not complement mutation of *ocd*[6]
sma1844	Aminobutyraldehyde dehydrogenase	Too general of an annotation in the *S. meliloti* 1021 genome annotation
sma1155	magnesium transport via ABC system	Too general of an annotation in the *S. meliloti* 1021 genome annotation
sma0959	3oxoacylacylcarrierprotein reductase (nC10:0)	Too general of an annotation in the *S. meliloti* 1021 genome annotation
sma0958	acetolactate synthase	Does not complement auxotrophy of a *smc01431* mutation[3]
sma0956	glutamate1semialdehyde aminotransferase	Too general of an annotation in the *S. meliloti* 1021 genome annotation
sma0486	ornithine cyclodeaminase	Does not complement mutation of *ocd*[6]

Supplementary Table 2. Biomass composition used in this study

Component	Percent dry mass	Composition - % [a]
DNA [b]	2.8	Guanine - 31.05<br>Cytosine - 31.05<br>Adenine - 18.95<br>Thymine - 18.95
RNA [c]	7.1	Guanine - 28.09<br>Cytosine - 28.09<br>Adenine - 21.91<br>Uracil - 21.91
Protein [d]	49.3	Lysine - 3.20<br>Alanine - 12.01<br>Leucine 10.19<br>Phenylalanine - 3.94<br>Arginine 7.33<br>Glutamine - 2.90<br>Glycine - 8.46<br>Methionine - 2.44<br>Valine - 7.58<br>Proline - 5.03<br>Tyrosine - 2.29<br>Aspartate - 5.31<br>Glutamate - 5.84<br>Histidine - 2.11<br>Threonine - 5.15<br>Cysteine - 0.93<br>Isoleucine - 5.48<br>Tryptophan - 1.38<br>Asparagine - 2.64<br>Serine - 5.80
Lipid [e]	12.8	PG(36:2) - 7.82<br>CL(36:2) - 3.11<br>PE(36:2) - 25.35<br>PC(36:2) - 59.92<br>SL(36:2) - 2.00<br>OL(36:1) - 1.80
PHB	17.6	N/A
Glycogen	0.4	N/A
LPS	3	N/A
Cell wall	2	N/A
LMW Succinoglycan [f]	4 [g]	N/A
HMW Succinoglycan [f]	1 [g]	N/A
Putrescine	Trace	N/A
Sperimdine	Trace	N/A
Nod factor [h]	1 mg per gm cell dry weight	N/A

[a] Where applicable, the subunit composition of each macromolecule is given, as is what percentage of the macromolecule that each subunit accounts for.

[b] Composition based on the overall GC content of *S. meliloti*[1].

[c] The GC content for mRNA was estimated from the overall GC content of *S. meliloti*[1]. The GC content of tRNA was estimated based on the GC content of the 10 most common codons in *S. meliloti*[1,7]. The GC content of rRNA was determined based on the *rrn* loci of *S. meliloti*[1]. The

overall composition of cellular RNA was determined assuming 80% rRNA, 15% tRNA, and 5% mRNA.

[d] The amino acid composition was estimated based on the codon usage of *S. meliloti*[7].

[e] The lipid composition used was as previously determined for *S. meliloti*, with each lipid class represented by a single lipid of the most common lipid size[8-11].

[f] A 4:1 ratio of low molecular weight (LMW) to high molecular weight (HMW) succinoglycan was set as previously determined[12].

[g] These numbers are for when growth was modelled in bulk soil. When growth was simulated in the rhizosphere, the amount of both LMW and HMW succinoglycan was doubled.

[h] Nod factor was included in the biomass composition when growth was modelled in the rhizosphere, but not when growth was modelled in bulk soil.

Supplementary Table 3. Summary of the fitness effect of individual gene deletions*

Replicon	Niche	Non-essential†	Essential†	Fitness contributing†
Chromosome	Bulk soil	704 (74.6)	191 (20.2)	49 (5.2)
	Rhizosphere	702 (74.4)	193 (20.4)	48 (5.1)
	Nodule	882 (93.6)	59 (6.3)	1 (0.1)
pSymB	Bulk soil	369 (94.9)	15 (3.9)	5 (1.3)
	Rhizosphere	356 (91.6)	15 (3.9)	18 (4.6)
	Nodule	388 (99.7)	0 (0.0)	1 (0.3)
pSymA	Bulk soil	240 (100)	0 (0.0)	0 (0.0)
	Rhizosphere	232 (96.7)	8 (3.3)	0 (0.0)
	Nodule	230 (95.8)	10 (4.2)	0 (0.0)

* Values indicate the number of genes (% of genes from the given replicon). † Non-essential – fitness of mutant > 99% of the wild type; Essential – fitness of mutant < 1% of the wild type; Fitness contributing – fitness of mutant ≥ 1% and ≤ 99% of the wild type.

Supplementary Table 4. The location of transcription factors regulating genes associated with environmental variable and fitness determining reactions.

Replicon	Entire Genome	Bulk Fit	Rhizo Fit	Nodule Fit	B2R More	B2R Less*	R2N More	R2N Less*
Chromosome	0.488 (312)	0.500 (1)	0.333 (1)	0.000 (0)	0.667 (6)	0.259 (7)	0.619 (13)	0.184 (7)
pSymB	0.353 (226)	0.500 (1)	0.667 (2)	1.000 (1)	0.333 (3)	0.741 (20)	0.143 (3)	0.632 (24)
pSymA	0.159 (102)	0.000 (0)	0.000 (0)	0.000 (0)	0.000 (0)	0.000 (0)	0.238 (5)	0.184 (7)

Values are ratios of all genes in the given group (column) with the gene count given in brackets. Replicon – the replicon on which the transcriptional regulator is located. Entire Genome – the distribution in the entire *S. meliloti* 1021 genome. Bulk Fit – genes whose deletion results in a fitness decrease in bulk soil. Rhizo Fit – genes whose deletion results in a fitness decrease in the rhizosphere. Nodule Fit – genes whose deletion results in a fitness decrease in the nodule. B2R More and B2R Less– genes associated with more important and less important reactions, respectively, following the bulk soil to rhizosphere transition. R2N More and R2N Less– genes associated with more important and less important reactions, respectively, following the rhizosphere to nodule transition. Statistically significant biases within each group, compared to the entire genome, was determined using Pearson's Chi Squared tests: * p-value < 0.001.

Supplementary Table 5. Summary of the pangenome classification of genes present in iGD1575.

Compartment	Genome	iGD1575*	Bulk Act*	Rhizo Act*	Nodule Act*	Bulk Fit*†	Rhizo Fit*†	Nodule Fit
Core	0.697 (4337)	0.827 (1296)	0.834 (637)	0.848 (6.38)	0.851 (344)	0.962 (51)	0.970 (64)	1.000 (2)
Accessory	0.285 (1775)	0.172 (269)	0.165 (126)	0.150 (113)	0.146 (59)	0.038 (2)	0.030 (2)	0.000 (0)
Unique	0.017 (106)	0.001 (2)	0.001 (1)	0.001 (1)	0.001 (1)	0.000 (0)	0.000 (0)	0.000 (0)

Values are ratios of all genes in the given group (column) with the gene count given in brackets. Compartment – the pangenome classifications. The distribution is shown for: the entire *S. meliloti* 1021 genome (entire genome), the model (iGD1575), genes associated with reactions active in bulk soil (Bulk Act), the rhizosphere (Rhizo Act), or the nodule (Nodule Act), and genes contributing to fitness in bulk soil (Bulk Fit), the rhizosphere (Rhizo Fit), or the nodule (Nodule Fit). * Distribution is significantly different (p-value < 0.001) from that of the *S. meliloti* 1021 genome as determined using Pearson's Chi Squared tests. † Distribution is significantly different (p-value < 0.05) from that of iGD1575 as determined using Pearson's Chi Squared tests.

Supplementary Table 6. Exchange reaction bounds for setting the environmental conditions

		Bulk Soil			Rhizosphere			Nodule		
Compound	Exchange reaction*	Lower Bound	Upper Bound	Flux rate	Lower Bound	Upper Bound	Flux rate	Lower Bound	Upper Bound	Flux rate
L-arabinose	EX_cpd00224_e0	-0.28889705	1000	-0.28889705	-0.26974143	1000	-0.26974143	0	1000	0
D-galactose	EX_cpd00108_e0	-0.25290619	1000	-0.25290619	-0.18317325	1000	-0.18317325	0	1000	0
D-glucose	EX_cpd00027_e0	-0.61972444	1000	-0.61972444	-0.04098397	1000	-0.04098397	0	1000	0
D-mannose	EX_cpd00138_e0	-0.2540567	1000	-0.2540567	-0.05436649	1000	-0.05436649	0	1000	0
L-rhamnose	EX_cpd00396_e0	-0.13913636	1000	-0.13913636	-0.02927426	1000	-0.02927426	0	1000	0
Xylose	EX_cpd00154_e0	-0.21280795	1000	-0.21280795	-0.04600242	1000	-0.04600242	0	1000	0
D-ribose	EX_cpd00105_e0	-0.01862144	1000	-0.01862144	0	1000	0	0	1000	0
Sucrose	EX_cpd00076_e0	0	1000	0	-0.02049199	1000	-0.02049199	0	1000	0
D-raffinose	EX_cpd00382_e0	0	1000	0	-0.01024599	1000	-0.01024599	0	1000	0
Stachyose	EX_cpd01133_e0	0	1000	0	-0.03073798	1000	-0.03073798	0	1000	0
Succinate	EX_cpd00036_e0	-0.0056245	1000	-0.0056245	-0.12240603	1000	-0.12240603	-1.326	0	-1.326
L-malate	EX_cpd00130_e0	-0.03649016	1000	-0.03649016	-0.79413596	1000	-0.79413596	-1.1122	1000	-1.1122
L-aspartate	EX_cpd00041_e0	0	1000	0	-0.03205557	1000	-0.03205557	0	1000	0
L-threonine	EX_cpd00161_e0	-0.0068175	1000	-0.0068175	-0.00961667	1000	-0.00961667	0	1000	0
L-serine	EX_cpd00054_e0	0	1000	0	-0.01004986	1000	-0.01004986	0	1000	0
L-glutamate	EX_cpd00023_e0	0	1000	0	-0.0283302	1000	-0.0283302	-2	10	-2
L-proline	EX_cpd00129_e0	-0.01102953	1000	-0.01102953	0	1000	0	0	1000	0
Glycine	EX_cpd00033_e0	-0.0068175	1000	-0.0068175	-0.00693094	1000	-0.00693094	0	1000	0
L-alanine	EX_cpd00035_e0	-0.02780553	1000	-0.02780553	-0.00823049	1000	-0.00823049	0	1000	0
L-valine	EX_cpd00156_e0	-0.02928849	1000	-0.02928849	-0.00337883	1000	-0.00337883	0	1000	0
L-cysteine	EX_cpd00084_e0	-0.0068175	1000	-0.0068175	0	1000	0	0	1000	0
L-isoleucine	EX_cpd00322_e0	-0.0199273	1000	-0.0199273	-0.00186269	1000	-0.00186269	0	1000	0
L-leucine	EX_cpd00107_e0	-0.03596182	1000	-0.03596182	-0.00303228	1000	-0.00303228	0	1000	0
L-tyrosine	EX_cpd00069_e0	-0.0068175	1000	-0.0068175	-0.00233919	1000	-0.00233919	0	1000	0
GABA	EX_cpd00281_e0	0	1000	0	0	1000	0	0	1000	0
L-ornithine	EX_cpd00064_e0	-0.0068175	1000	-0.0068175	-0.00831712	1000	-0.00831712	0	1000	0
L-histidine	EX_cpd00119_e0	-0.0068175	1000	-0.0068175	-0.00506825	1000	-0.00506825	0	1000	0
L-arginine	EX_cpd00051_e0	-0.0068175	1000	-0.0068175	-0.00948672	1000	-0.00948672	0	1000	0
Trans-4-hydroxy-l-proline	EX_cpd00851_e0	0	1000	0	-0.26974144	1000	-0.26974144	0	1000	0
Ammonia	EX_cpd00013_e0	-7.5	1000	-7.5	-7.5	1000	-7.5	0	1000	0
Nitrate	EX_cpd00209_e0	-7.5	1000	0	-7.5	1000	0	0	1000	0
Sulfate	EX_cpd00048_e0	-15	1000	-0.05219940	-15	1000	-0.05604370	-1000	1000	-0.01900238
Phosphate	EX_cpd00009_e0	-15	1000	-0.17306777	-15	1000	-0.14804998	-1000	0	-0.01425178
H_2O	EX_cpd00001_e0	-15	1000	19.38238966	-15	1000	16.74436329	-1000	1000	2.53455439
O_2	EX_cpd00007_e0	-15	1000	-0.06956818	-15	1000	-0.12160182	-1.26	1000	-0.01187648

Continued on the next page

		Bulk Soil			Rhizosphere			Nodule		
Compound	Exchange reaction	Lower Bound	Upper Bound	Flux rate	Lower Bound	Upper Bound	Flux rate	Lower Bound	Upper Bound	Flux rate
CO_2	EX_cpd00011_e0	-15	1000	-5.60835703	-15	1000	-4.83015412	-1000	1000	6.05032969
Mn_2	EX_cpd00030_e0	-15	1000	0	-15	1000	0	0	1000	0
Zn_2	EX_cpd00034_e0	-15	1000	0	-15	1000	0	0	1000	0
Cu_2	EX_cpd00058_e0	-15	1000	0	-15	1000	0	0	1000	0
Ca_2	EX_cpd00063_e0	-15	1000	0	-15	1000	0	0	1000	0
H^+	EX_cpd00067_e0	-15	1000	-15	-15	1000	-15	-1000	1000	-12.54547743
Cl^-	EX_cpd00099_e0	-15	1000	0	-15	1000	0	0	1000	0
Biotin	EX_cpd00104_e0	-15	1000	0	-15	1000	0	0	1000	0
Co_2	EX_cpd00149_e0	-15	1000	0	-15	1000	0	0	1000	0
K^+	EX_cpd00205_e0	-15	1000	0	-15	1000	0	0	1000	0
Mg^{2+}	EX_cpd00254_e0	-15	1000	0	-15	1000	0	-1000	1000	-0.00475059
Na^+	EX_cpd00971_e0	-15	1000	0	-15	1000	0	0	1000	0
Fe^{2+}	EX_cpd10515_e0	-15	1000	0	-15	1000	0	-1000	1000	-0.02375297
Fe^{3+}	EX_cpd10516_e0	-15	1000	0	-15	1000	0	0	1000	0
Cob(I)alamin	EX_cpd00635_e0	0	1000	0	0	1000	0	-1000	1000	-0.00475059
Homocitrate	EX_cpd00919_e0	0	1000	0	0	1000	0	-1000	1000	-0.00475059
Molybdate	EX_cpd11574_e0	0	1000	0	0	1000	0	-1000	1000	-0.00475059
Thiamine	EX_cpd00305_e0	0	1000	0	0	1000	0	-1000	1000	-0.00475059
M-inositol	EX_cpd00121_e0	0	1000	0	0	1000	0	-0.01	0	-0.01
N_2	EX_cpd00528_e0	0	1000	0	0	1000	0	-1000	0	-0.47505938
Urea	EX_cpd00073_e0	0	1000	2.5546521	0	1000	2.89929159	0	1000	0
Biomass	EX_cpd11416_c0	0	1000	0.32084806	0	1000	0.25948388	0	1000	0
5-Methylthioadenosine	EX_cpd00147_e0	0	1000	0.00001692	0	1000	0.00001369	0	1000	0
H_2	EX_cpd11640_e0	0	1000	0	0	1000	0	0	1000	0.47505938
Fixed NH_3	EX_cpdFixed_e0	0	1000	0	0	1000	0	0	1000	0.47505938

*If an exchange reaction is not listed, the lower boundary was set to '0', the upper boundary set to '1000', and the reaction did not carry flux in any of the three conditions.

Supplementary Table 7. Single copy iGD1575 genes essential for growth in M9-sucrose minimal medium

pSymA
None
pSymB
smb21690, smb21327, smb21324, smb20961, smb20959, smb20958, smb20957, smb20956, smb20954, smb20949, smb20948, smb20946, smb20944, smb20943, smb20652
Chromosome
smc04434, smc04410, smc04406, smc04405, smc04346, smc04320, smc04268, smc04213, smc04088, smc04045, smc04003, smc04002, smc04001, smc03990, smc03934, smc03925, smc03885, smc03881, smc03863, smc03861, smc03859, smc03858, smc03856, smc03826, smc03823, smc03807, smc03797, smc03795, smc03772, smc03770, smc03173, smc03172, smc03112, smc02912, smc02905, smc02899, smc02898, smc02837, smc02804, smc02802, smc02790, smc02782, smc02767, smc02766, smc02765, smc02755, smc02725, smc02717, smc02700, smc02692, smc02686, smc02652, smc02574, smc02572, smc02570, smc02569, smc02568, smc02567, smc02551, smc02496, smc02450, smc02438, smc02305, smc02245, smc02165, smc02124, smc02113, smc02101, smc02099, smc02093, smc02091, smc02089, smc02080, smc02076, smc02073, smc02070, smc02069, smc02064, smc01934, smc01880, smc01878, smc01875, smc01871, smc01868, smc01867, smc01866, smc01864, smc01863, smc01862, smc01861, smc01804, smc01803, smc01801, smc01761, smc01756, smc01732, smc01726, smc01720, smc01567, smc01563, smc01461, smc01444, smc01431, smc01430, smc01369, smc01364, smc01360, smc01353, smc01352, smc01350, smc01343, smc01321, smc01320, smc01319, smc01318, smc01317, smc01316, smc01314, smc01313, smc01310, smc01309, smc01308, smc01307, smc01306, smc01305, smc01304, smc01303, smc01302, smc01301, smc01300, smc01299, smc01298, smc01297, smc01296, smc01295, smc01294, smc01293, smc01292, smc01291, smc01290, smc01287, smc01286, smc01285, smc01283, smc01233, smc01231, smc01209, smc01192, smc01189, smc01161, smc01152, smc01127, smc01121, smc01116, smc01109, smc01100, smc01096, smc01027, smc01025, smc01018, smc01010, smc01004, smc00993, smc00919, smc00918, smc00917, smc00908, smc00892, smc00855, smc00851, smc00723, smc00711, smc00704, smc00696, smc00695, smc00659, smc00643, smc00615, smc00614, smc00595, smc00568, smc00567, smc00565, smc00554, smc00552, smc00551, smc00526, smc00508, smc00495, smc00494, smc00493, smc00488, smc00485, smc00475, smc00421, smc00415, smc00414, smc00412, smc00408, smc00394, smc00385, smc00366, smc00365, smc00364, smc00363, smc00335, smc00334, smc00333, smc00323, smc00296, smc00293, smc00236, smc00235, smc00232, smc00155, smc00007

Supplementary Table 8. *Sinorhizobium meliloti* strains used in this study.

Strain	Relevant characteristics	Reference
RmP110	Wild type Rm1021 (SU47 *str-21*) with fixed *pstC*; Sm^R	13
RmP790	RmP110, ΔB108 (1,131,168-1,169,073); Sm^R Nm^R Gm^R Tc^R	14
RmP799	RmP110, ΔB109 (1,180,466-1,204,770); Sm^R Tc^R	14
RmP811	RmP110, ΔB118 (1,323,078-1,528,150); Sm^R Tc^R	14
RmP876	RmP110, ΔB141 (101,396-466,499); Sm^R Tc^R	14
RmP1055	RmP110, ΔB161 (1,679,723-49,523); Sm^R Tc^R	14
RmP1059	RmP110, ΔB154 (62,137-100,636); Sm^R Nm^R Gm^R Tc^R	14
RmP2712	RmP110, ΔB179 (1,207,052–1,322,226); Sm^R Sp^R Nm^R Gm^R	15
RmP2715	RmP110, ΔB182 (1,529,711-1,677,882); Sm^R Nm^R Gm^R Tc^R	14
RmP2716	RmP110, ΔB181 (870,505-1,129,758); Sm^R Nm^R Gm^R Tc^R	14
RmP2717	RmP110, ΔB163 (451,557-651,863); Sm^R Nm^R Gm^R Tc^R	14
RmP2754	RmP110, ΔB180 (635,940-869,642); Sm^R Nm^R	14

Sm – streptomycin; Nm – neomycin; Gm – gentamicin; Tc – tetracycline; Sp – spectinomycin

SUPPLEMENTARY NOTES

Supplementary Note 1. Prediction of the genetic basis of D-galacactosamine metabolism. The genetic basis for several of the observed carbon utilization phenotypes has not been previously examined in *S. meliloti*. By combining our mutant phenotype data with iGD1575, the output of the DuctApe software[16], the *S. meliloti* genome annotation[1], and a previously published ABC transporter induction study[17], we were able to predict novel carbon catabolic loci. One example compound is D-galactosamine. D-galactosamine was not present in the Phenotype MicroArray^{TM}, but *N*-acetyl-D-galactosamine, whose catabolism proceeds via D-galactosamine, was tested. Both the ΔB180 and ΔB181 deletion mutants failed to utilize *N*-acetyl-D-galactosamine as a carbon source. It was previously shown that two ABC transporters within ΔB180, one encoded by *smb21135-smb21138* and the second by *smb21216, smb21219-smb21221*, are induced by D-galactosamine[17]. The *smb21216, smb21219-smb21221* operon also includes a putative sugar isomerase (*smb21218*) and sugar amine kinase (*smb21217*). Thus, we hypothesize that the Smb21216, Smb21219-Smb21221 transporter, as well as potentially the Smb21135-Smb21138 transporter, transports D-glucosamine, which is phosphorylated by Smb21217 to D-glucosamine-6-phosphate, and converted to D-tagatose-6-phosphate by Smb21218. D-tagatose-6-phosphate can then be further metabolized via two steps to glyceraldehyde-3-phosphate and dihydroxyacetone phosphate, both which can enter glycolysis. At least one of these two final steps is located within ΔB181 as this mutant cannot grow with *N*-acetyl-D-glucosamine or D-tagatose. In fact, the *smb21373-smb21377* operon, removed in ΔB181, encodes an ABC transporter that we predict transports D-tagatose and that indeed is induced by tagatose[17], as well as a putative carbohydrate kinase and a putative D-tagatose-1,6-bisphosphate aldolase that could complete the D-galactosamine catabolic pathway.

Supplementary Note 2. Validation of the metabolic switching detection during niche adaptation. Flux variability analysis (FVA) was employed to ensure that the observed flux changes between environments were true changes. During FBA, one solution of potentially multiple solutions giving the same optimal flux through the objective function is returned. FVA examines the range of flux that can possibly be carried by each reaction while still maintaining optimal flux through the objective function.

A total of 199 reactions were considered to change in importance between simulated growth in bulk soil and the rhizosphere based on changes in flux and/or the fitness contribution. Of these, 63 (31.7%) are supported by fitness data while 136 (68.7%) are classified as changing solely on the basis of flux. Of the 136 reactions, the predicted reaction flux during growth in the rhizosphere for 61 of them was outside the FVA range for the reaction when grown in bulk soil. This supports that optimal biomass production in the rhizosphere required that flux through these 61 reactions changed. To further examine the remaining 75 reactions, where the reaction flux in the rhizosphere was within the FVA range of the reaction when grown in bulk soil, the flux through each reaction was individually set to the rhizosphere value, and the flux distribution during growth in bulk soil was monitored. In all but two cases, doing so caused the flux through at least one other reaction (with an average of 9 reactions and standard deviation of 4) to move outside of the rhizosphere FVA range of that reaction. Or in other words, while 61 of the predicted changing reactions between bulk soil and rhizosphere could be artificially set to the rhizosphere flux rate without impairing growth in bulk soil, in all but 2 cases, doing so would

require an average of 9 new changes following transition from growth in bulk soil to the rhizosphere. Additionally, when flux through all 61 of the reactions were simultaneously set to the corresponding flux value from the rhizosphere, and growth simulated in bulk soil, the model became unsolvable. Together, these data support that 197 of the 199 reactions (99.0%) predicted to change in importance during transition between growth in bulk soil and the rhizosphere are true changes.

The same procedure was used to confirm the flux changes observed during transition between the rhizosphere and the nodule environments. Of the 451 reactions that were considered changing, 292 (64.7%) were supported by fitness data while 159 (35.3%) are based solely on flux data. Of these 159 reactions, the predicted flux through 57 in the nodule was outside the FVA range for the reaction during simulated growth in the rhizosphere. For all the remaining 102 reactions, individually setting the flux during growth in the rhizosphere to the flux value predicted in the nodule resulted in the flux through at least one (average of 4.5, standard deviation 2) other reaction to move outside the nodule FVA range of that reaction. Additionally, the model becomes unsolvable in the rhizosphere when flux through all 102 reactions are simultaneously set to the corresponding nodule flux value. When considered together, these data support that all of the reactions predicted to change in importance during transition between growth in the rhizosphere and nitrogen fixation in the nodule are true changes.

Supplementary Note 3. Robustness with respect to changes in the nutrients composition and uptake rates. We explored the effect of random variations in the composition and uptake rates of the nutrients present in each of the simulated ecological niches (bulk soil, rhizosphere, and nodule) on the main outcomes of the model, i.e. predicted growth rates, number of essential and fitness contributing genes and gene pairs. Results obtained (shown in Supplementary Figures 7 and 8) revealed that, overall, conclusions drawn concerning the role of pSymA and pSymB throughout the *S. meliloti* lifecycle and the growth phenotypes in different conditions are not influenced by such variations. In all environments, the number of essential genes shows very little variation across all the iterations, and the number of essential plus fitness contributing genes on each replicon is also predominately unaffected by the environmental variations. This trend is observed also in the case of essential and fitness contributing gene-pairs (Supplementary Figure 8). A notable exception is represented by the effectiveness of the symbiosis in the nodule (Supplementary Figure 7), which seems to be more dependent on the composition and the utilization rate of the input compounds. However, this higher variation is not surprising as a limited number of nutrients that are used by the bacteria are available to the bacteroid in the nodule; as such, any change in one nutrient is likely to influence flux through the objective function much more.

Overall, the stability of the number of essential/fitness contributing genes across all permutations confirms that the presented results are robust and supports that the conclusions drawn in this work.

Supplementary Note 4. Few biases were detected in the genomic localization of the transcriptional regulators of metabolic genes. When compared to a recent regulon analysis in *S. meliloti*[2], even if not conclusive due to the low representation in terms of gene numbers, a bias was observed for genes associated with reactions classified as less important during either the bulk soil to rhizosphere or the rhizosphere to nodule transitions to be regulated by a pSymB encoded transcription factor (Supplementary Table 4 and Supplementary Data 5). However,

while this may be suggestive of a bias for the regulatory machinery associated with niche adaptation to be encoded by pSymB, it may also simply reflect a bias in the dataset as most of the regulated genes were located on pSymB and transcription factors tend to regulated genes on their own replicon[2].

Supplementary Note 5. The core *S. meliloti* genome is over-represented among the fitness contributing genes. An advantage of using *S. meliloti* as a model organism is that many strains have been fully sequenced, facilitating the study of the *S. meliloti* pangenome. We classified all genes associated with the environmentally variable or fitness promoting reactions as belonging to the 'core', 'accessory', or 'unique' genome (Supplementary Data 5) based on the results of Galardini *et al.*[2]. Perhaps not surprisingly given that iGD1575 is enriched in the core metabolic processes relative to the entire *S. meliloti* genome, the core genome was over-represented in the model (Supplementary Table 5). Remarkably, however, nearly all genes contributing to fitness in either bulk soil, the rhizosphere, or the nodule belonged to the core genome, a clear enrichment relative to the percentage of core genes in iGD1575 overall. This observation highlights that metabolic genes contributing to the fitness of the cell are highly likely to be or become part of the core genome, emphasizing the functional role of core genome as common tool set of genes for a given bacterial species.

Supplementary Note 6. Identification of essential model genes. Currently, no high throughput systematic knock-out studies of *S. meliloti* exist in the literature, and so the complete set of essential genes in this organism is not known. However, previous work has shown that only two essential genes exist on the pSymB chromid and none are present on pSymA[15,18,19]. In this work we examined a library of pSymB deletion mutants that collectively remove 98% of pSymB[14] for carbon utilization phenotypes using Phenotype MicroArrays™. We also replicated this experiment *in silico* using iGD1575. In order to do so, it was necessary to ensure that none of the *in silico* deletion mutants were lethal. To test this, we used an *in silico* single gene deletion analysis to identify single copy essential genes in iGD1575 when grown in a minimal medium with sucrose as the carbon source and with thiamine supplementation[18,19]. In this context, an essential gene refers to a gene whose deletion prevents the formation of at least one biomass precursor when grown. This analysis identified a total of 231 single copy essential metabolic genes (Supplementary Table 8), none of which are on pSymA. However, of the 231 essential genes, 216 were on the chromosome while 15 were on pSymB.

Of the 15 genes on pSymB, 12 are involved in the biosynthesis of succinoglycan. While these genes are not truly essential, the inclusion of succinoglycan in the biomass formation reaction resulted in them being considered essential in the model. Two of the essential pSymB genes, *wgaG* and *wgaJ*, are predicted to be required for lipopolysaccharide (LPS) biosynthesis. Further examination, through a double deletion analysis and manual screening, revealed another two reactions associated with multiple redundant pSymB genes that are essential for LPS synthesis. All four reactions were required for the synthesis of dTDP-rhamnose, necessary for the production of the O-antigen. Little is known about LPS biosynthesis in *S. meliloti*, and it is possible that these genes are truly required for complete LPS synthesis. But whereas *Sinorhizobium* LPS mutants that cannot encorporate rhamnose into their LPS produce a truncated LPS yet survive[20], the rigidity of the model and encorporation of complete LPS in the biomass reaction meant that such a mutant would fail to produce biomass *in silico*. Finally, *ansB* was not surprisingly determined to be essential as it is the only *S. meliloti* gene predicted to be involved

in asparagine biosynthesis, although experimental evidence indicates it is not essential for asparagine biosynthesis and the asparagine biosynthetic pathway in *S. meliloti* remains unidentified[18]. The two genes on pSymB that are truly essential are a tRNA and a protein involved in ribosomal biogenesis (*engA*)[15], and are therefore not present in the model.

When succinoglycan is removed from the biomass reaction, and unknown GPRs are added to the AsnB and the four dTDP-rhamnose synthesis reactions, the simultaneous removal of all pSymA and pSymB genes from the model does not prevent biomass formation. Thus, for the *in silico* experiments testing the metabolic capacity of iGD1575, the model was modified as described in the previous sentence so that all deletion mutants were viable.

SUPPLEMENTARY METHODS

Metabolic network reconstruction. A draft metabolic model was constructed using the KBase Narrative Interface (www.kbase.us). The *S. meliloti* 1021 annotated genome was imported from the public KBase database, and a draft metabolic model was reconstructed using the 'Build metabolic model' method. This draft model was gap-filled using the 'Gapfill metabolic model' method to allow biomass formation when grown in a minimal medium containing metal ions, succinate, ammonium, sulphate, phosphate, and biotin. The model was then downloaded, the KBase annotations in the 'Gene_association' field replaced with the *S. meliloti* 1021 locus tags, and the KBase annotations in the 'Protein_association' field replaced with the *S. meliloti* 1021 gene names[1]. The KBase biomass objective was removed, new objective functions were formulated (representing biomass formation and symbiosis, as described below) and the model manually gap filled to produce flux through each of the objective functions.

Manual curation and further expansion of the model through the inclusion of additional reactions and gene-protein-reaction associations (GPRs) was performed in several main stages. Where possible, 'Unknown' GPRs were replaced with genes from the *S. meliloti* 1021 genome, and when supported by published experimental data, incorrect GPRs and reactions were removed from the draft model. We next identified and included additional metabolic and transport genes in the *S. meliloti* 1021 genome annotation. Following this, the genes present in the draft model were compared to the list of genes included in the existing *S. meliloti* metabolic model iHZ565[21], and the majority of the additional genes and associated reactions from iHZ565 were added to our draft model. However, 31 genes were not transferred from iHZ565 (Supplementary Table 1) as the annotation was extremely general and we lacked confidence in the true substrates/products of the reaction, experimental data was inconsistent with their inclusion, or the reaction produced a dead-end metabolite (a metabolite produced or consumed by only a single reaction, meaning that the reaction will never be active during flux balance analysis). An iterative gap-filling procedure was then employed to reconcile the predictions of 'growth' or 'no growth' with various carbon and nitrogen substrate with the known ability or inability of *S. meliloti* 1021 to grow on these substrates. Most of the experimental growth data came from a previous Phenotype MicroArray™ (Biolog) experiment[22], with a few substrate taken from additional literature sources[23-26]. Finally, a library of large *S. meliloti* deletion mutants was screened for carbon utilization defects using the Omnilog Phenotype MicroArray™ system (Biolog), as described below. *In silico* predictions were compared with the experimental results, and were necessary and possible, discrepancies were fixed by further refinement and expansion of the metabolic model. Where possible, GPRs for transport reactions were added based on published mutation or induction studies; otherwise, transport reactions were added as a diffusion reaction with no associated GPR. Support for manually added transporters and metabolic reactions came from experimental evidence, review articles, and the KEGG and BioCyc databases[3-6,8,9,17,22,23,25-65].

The final *S. meliloti* model was termed iGD1575 in accordance with the nomenclature standard[66], and includes 1575 genes, 1825 reactions, and 1579 metabolites. The SBML file of the model was validated by the online SBML validator tool (http://sbml.org/Facilities/Validator/), and is available as Supplementary Data 6.

Metabolic modelling. The ability of the model to produce flux through the specified objective functions was examined using flux balance analysis (FBA). Simulations were

performed in Matlab R2015a (Mathworks), using scripts from the Cobra Toolbox[67] and the Gurobi 6.0.1 solver (www.gurobi.com). Single gene deletion and double gene deletion analyses were performed using methods in the Cobra Toolbox. Iteratively removing each reaction from the model and then examining the effect with FBA determined the essentiality of each individual reaction. Metabolic capacity was determined *in silico* by iteratively providing the model with a unique carbon or nitrogen source and then observing the ability of the model to produce biomass with FBA. The predicted effect of the large-scale genome deletions on the growth phenotype (either growth or no growth) was addressed by simultaneously removing all reactions dependent on the deleted genes from the model and then running the *in silico* phenotype microarray experiment. For this analysis, exopolysaccharide was removed from the objective function and an unknown GPR added to the other four essential reactions dependent on pSymB genes (see Supplementary Note 6 for additional details) in order to allow all deletion mutants to grow in the minimal media.

Evaluation of robustness with respect to changes in the nutrients composition and uptake rates. Simulation of growth/symbiosis in 1000 randomly generated media for each environment was performed as follows. For each of the 1000 iterations, a random variation in the allowable uptake rate for each of the nutrients in the medium was introduced, with the allowable variation set to a value 50% greater or lower than the original one (e.g. if the original uptake rate was 1 mmol g^{-1} h^{-1}, in each of the 1000 conditions, the uptake rate was randomly set to a speed between 0.5 to 1.5 mmol g^{-1} h^{-1}). Additionally, further noise was introduced by randomly removing, at each iteration, two nutrients from the niche simulated nutrients set and restoring them for the following iteration. FBA was then used, at each iteration, to evaluate i) the predicted growth rate in each of the iterations for each of the three environments, ii) the variations in the number of essential genes, and iii) the variations in replicon specific essential plus fitness contributing genes. Due to time constraints, when determining the variation in the number of replicon specific essential/fitness-contributing gene pairs related to random changes in the nutrients composition, the number of iterations was reduced to 100.

Flux visualization. Metabolic networks were visualized with the online tool iPath 2.0[68]. Where possible, KEGG IDs were associated with each reaction based on comparison with the Seed Reference list, and these reactions mapped to the corresponding reaction, if one existed, in the 'Metabolic Pathway' map of iPath.

***In silico* environmental representations.** *In silico* representations of the nutritional composition of the rhizosphere and bulk soil were constructed from data available in the literature. For both the rhizosphere and bulk soil *in silico* representations, ammonium and nitrate were included at a one to one ratio, and sufficient ammonium, nitrate, phosphate, sulphate, metal ions, and gases were included so that these compounds were not growth rate limiting.

The sugar content of the *in silico* rhizosphere was primarily set according to the average monosaccharide composition of pea (*Pisum sativum*) and cowpea (*Vigna unguiculata*) root mucilage[69,70]. Sucrose, raffinose, and stachyose were added to the list of sugars, with the ratio of these three sugars set according to their approximate ratio in alfalfa (*Medicago sativa*) root exudate[71]. The total amount of sucrose, raffinose, and stachyose was arbitrarily set so that 60% of the total glucose was within these sugars, and the amounts of free glucose and galactose were reduced accordingly. The organic acids included in the rhizosphere, and their relative ratios, was

based on their prevalence in unstressed alfalfa root exudate[72]. The total ratio of organic acids to sugars in legume root exudate was not found; however, organic acids were ~ 10, 4, and 2 fold more prevalent than sugars in the root exudates of tomato (*Lycopersicon esculentum*), cucumber (*Cucumis sativus*), and sweet pepper (*Capsicum annum*), respectively[73]. Therefore, the total amount of organic acid in the rhizosphere was set at a molar ratio two fold greater than the total carbohydrates. The amino acid content of the rhizosphere was primarily based on the amount of each amino acid present in pea root exudate when grown in quartz sand[74]. To this, hydroxyproline was added according to the serine to hydroxyproline ratio in pea root mucilage[69]. The carbohydrate to amino acid ratio in legume root exudate was not found; however, an approximate carbohydrate to protein ratio of four to one, or greater, was observed in three rice varieties (*Oryza sativa*)[75]. Therefore, the total amount of amino acids in the rhizosphere was set at a molar ratio four fold less than the total carbohydrates.

The molar ratio of sugars in the *in silico* bulk soil representation was set as determined previously[76]. Unlike in the rhizosphere condition, sucrose, raffinose, and stachyose were not added to the bulk soil as they appear to be largely absent[71,77]. The concentration of organic acids appears to be quite low in bulk soil, with the organic acid content of the rhizosphere likely at least 50-fold higher than that of bulk soil[78]. Therefore, the three organic acids included in the rhizosphere formulation were also included in bulk soil, but at 2% the concentration. The dominant amino acid content of bulk soil was set as the average of two previously analyzed soil samples[79]. These amino acids accounted for 66.9% of the amino acids, with the remaining 33.1% split evenly between the non-modified amino acids not displayed in the reference[79] as they never exceeded 5% of the total amino acid population. The total carbohydrate content of the bulk soil was set ten fold higher than the total amino acid content, as determined previously[79].

The nutritional environment within the root nodule was set as used previously for constraint based modelling of iHZ565[21]. The upper and lower bounds for the exchange reactions in all three environments are listed in Supplementary Table 6.

Phenotype MicroArray™ analysis. *S. meliloti* RmP110[13], a derivative of *S. meliloti* 1021 in which a frameshift mutation within *pstC* was fixed, was used as the wild type reference strain. All deletion mutant strains were described previously[14,15], and consist of large deletions of the pSymB replicon that each span ~ 40 to 370 kilobase pairs. Phenotype MicroArray™ experiments were performed largely as described previously[22], using Biolog plates PM1 and PM2A. Strains were initially grown at 30°C on LBmc agar supplemented with $CoCl_2$[18]. To begin the Phenotype MicroArray™ analysis, colonies were picked up with sterile cotton swabs and resuspended in 0.8% NaCl to a cell density of 81% turbidity (OD_{600} ~ 0.1) as measured with a Biolog turbidimeter. 2 mL of each suspension was diluted in 22 mL of carbon free M9 minimal medium[18] containing 240 µL of the redox dye MixA 100x (Biolog), and 100 µL of the final mixture was added to each well of the Biolog plates. The one exception was *S. meliloti* RmP2754 (ΔB180), which grew very poorly when inoculated directly from the agar plate. Therefore, this strain and a second replicate of *S. meliloti* RmP110 were pregrown in liquid M9-glucose, washed twice with 0.8% saline, and diluted in 0.8% saline to a turbidity of 81%. This cellular suspension was then treated as described above and used to inoculate the PM plates. All PM plates were incubated at 30°C in an OmniLog plate reader, which measured reduction of the dye every 15 minutes for 120 hours.

SUPPLEMANTARY REFERENCES

1. Galibert, F. *et al.* The composite genome of the legume symbiont *Sinorhizobium meliloti*. *Science* **293,** 668–672 (2001).
2. Galardini, M. *et al.* Evolution of intra-specific regulatory networks in a multipartite bacterial genome. *PLOS Comput Biol* **11,** e1004478 (2015).
3. las Nieves Peltzer, de, M. *et al.* Auxotrophy accounts for nodulation defect of most *Sinorhizobium meliloti* mutants in the branched-chain amino acid biosynthesis pathway. *Mol Plant Microbe Interact* **21,** 1232–1241 (2008).
4. Harrison, J. *et al.* Glutathione plays a fundamental role in growth and symbiotic capacity of *Sinorhizobium meliloti*. *J. Bacteriol.* **187,** 168–174 (2005).
5. Geddes, B. A. & Oresnik, I. J. Genetic characterization of a complex locus necessary for the transport and catabolism of erythritol, adonitol and L-arabitol in *Sinorhizobium meliloti*. *Microbiology* **158,** 2180–2191 (2012).
6. diCenzo, G. C., Zamani, M., Cowie, A. & Finan, T. M. Proline auxotrophy in *Sinorhizobium meliloti* results in a plant-specific symbiotic phenotype. *Microbiology* **161,** 2341–2351 (2015).
7. Nakamura, Y., Gojobori, T. & Ikemura, T. Codon usage tabulated from international DNA sequence databases: status for the year 2000. *Nucleic Acids Res.* **28,** 292 (1999).
8. Weissenmayer, B., Gao, J. L., López-Lara, I. M. & Geiger, O. Identification of a gene required for the biosynthesis of ornithine-derived lipids. *Mol. Microbiol.* **45,** 721–733 (2002).
9. Gao, J.-L. *et al.* Identification of a gene required for the formation of lyso-ornithine lipid, an intermediate in the biosynthesis of ornithine-containing lipids. *Mol. Microbiol.* **53,** 1757–1770 (2004).
10. Zavaleta-Pastor, M. *et al.* *Sinorhizobium meliloti* phospholipase C required for lipid remodeling during phosphorus limitation. *Proc. Natl. Acad. Sci. U.S.A.* **107,** 302–307 (2010).
11. Basconcillo, L. S., Zaheer, R., Finan, T. M. & McCarry, B. E. A shotgun lipidomics study of a putative lysophosphatidic acid acyl transferase (PlsC) in *Sinorhizobium meliloti*. *Journal of Chromatography B* **877,** 2873–2882 (2009).
12. Glenn, S. A., Gurich, N., Feeney, M. A. & González, J. E. The ExpR/Sin quorum-sensing system controls succinoglycan production in *Sinorhizobium meliloti*. *J. Bacteriol.* **189,** 7077–7088 (2007).
13. Yuan, Z.-C., Zaheer, R. & Finan, T. M. Regulation and properties of PstSCAB, a high-affinity, high-velocity phosphate transport system of *Sinorhizobium meliloti*. *J. Bacteriol.* **188,** 1089–1102 (2006).
14. Milunovic, B., diCenzo, G. C., Morton, R. A. & Finan, T. M. Cell growth inhibition upon deletion of four toxin-antitoxin loci from the megaplasmids of *Sinorhizobium meliloti*. *J. Bacteriol.* **196,** 811–824 (2014).
15. diCenzo, G., Milunovic, B., Cheng, J. & Finan, T. M. The $tRNA^{arg}$ gene and *engA* are essential genes on the 1.7-mb pSymB megaplasmid of *Sinorhizobium meliloti* and were translocated together from the chromosome in an ancestral strain. *J. Bacteriol.* **195,** 202–212 (2013).
16. Galardini, M. *et al.* DuctApe: a suite for the analysis and correlation of genomic and OmniLog™ Phenotype Microarray data. *Genomics* **103,** 1–10 (2014).

17. Mauchline, T. H. *et al.* Mapping the *Sinorhizobium meliloti* 1021 solute-binding protein-dependent transportome. *Proc. Natl. Acad. Sci. U.S.A.* **103,** 17933–17938 (2006).
18. diCenzo, G. C., MacLean, A. M., Milunovic, B., Golding, G. B. & Finan, T. M. Examination of prokaryotic multipartite genome evolution through experimental genome reduction. *PLOS Genet* **10,** e1004742 (2014).
19. Oresnik, I. J., Liu, S. L., Yost, C. K. & Hynes, M. F. Megaplasmid pRme2011a of *Sinorhizobium meliloti* is not required for viability. *J. Bacteriol.* **182,** 3582–3586 (2000).
20. Ardissone, S., Noel, K. D., Klement, M., Broughton, W. J. & Deakin, W. J. Synthesis of the flavonoid-induced lipopolysaccharide of *Rhizobium* sp. strain NGR234 requires rhamnosyl transferases encoded by genes *rgpF* and *wbgA*. *Mol Plant Microbe Interact* **24,** 1513–1521 (2011).
21. Zhao, H., Li, M., Fang, K., Chen, W. & Wang, J. *In silico* insights into the symbiotic nitrogen fixation in *Sinorhizobium meliloti* via metabolic reconstruction. *PLOS ONE* **7,** e31287 (2012).
22. Biondi, E. G. *et al.* Metabolic capacity of *Sinorhizobium* (*Ensifer*) *meliloti* strains as determined by Phenotype MicroArray analysis. *Appl. Environ. Microbiol.* **75,** 5396–5404 (2009).
23. MacLean, A. M., White, C. E., Fowler, J. E. & Finan, T. M. Identification of a hydroxyproline transport system in the legume endosymbiont *Sinorhizobium meliloti*. *Mol Plant Microbe Interact* 1–12 (2009).
24. Boivin, C., Barran, L. R., Malpica, C. A. & Rosenberg, C. Genetic analysis of a region of the *Rhizobium meliloti* pSym plasmid specifying catabolism of trigonelline, a secondary metabolie present in legumes. *J. Bacteriol.* **173,** 2809–2817 (1991).
25. Chen, A.-M. *et al.* Identification of a TRAP transporter for malonate transport and its expression regulated by GtrA from *Sinorhizobium meliloti*. *Res. Microbiol.* **161,** 556–564 (2010).
26. Kohler, P. R. A., Choong, E. L. & Rossbach, S. The RpiR-Like repressor IolR regulates inositol catabolism in *Sinorhizobium meliloti*. *J. Bacteriol.* **193,** 5155–5163 (2011).
27. Geddes, B. A. & Oresnik, I. J. Inability to catabolize galactose leads to increased ability to compete for nodule occupancy in *Sinorhizobium meliloti*. *J. Bacteriol.* **194,** 5044–5053 (2012).
28. Jensen, J. B., Peters, N. K. & Bhuvaneswari, T. V. Redundancy in periplasmic binding protein-dependent transport systems for trehalose, sucrose, and maltose in *Sinorhizobium meliloti*. *J. Bacteriol.* **184,** 2978–2986 (2002).
29. Willis, L. B. & Walker, G. C. A novel *Sinorhizobium meliloti* operon encodes an α-glucosidase and a periplasmic-binding-protein-dependent transport system for α-glucosides. *J. Bacteriol.* **181,** 4176–4184 (1999).
30. Ampomah, O. Y. *et al.* The *thuEFGKAB* operon of rhizobia and *Agrobacterium tumefaciens* codes for transport of trehalose, maltitol, and isomers of sucrose and their assimilation through the formation of their 3-keto derivatives. *J. Bacteriol.* **195,** 3797–3807 (2013).
31. Geddes, B. A. & Oresnik, I. J. Physiology, genetics, and biochemistry of carbon metabolism in the alphaproteobacterium *Sinorhizobium meliloti*. *Can. J. Microbiol.* **60,** 491–507 (2014).
32. Dunn, M. F. Key roles of microsymbiont amino acid metabolism in rhizobia-legume interactions. *Critical Reviews in Microbiology* **41,** 411–451 (2015).

33. Kohler, P. R. A., Zheng, J. Y., Schoffers, E. & Rossbach, S. Inositol catabolism, a key pathway in *Sinorhizobium meliloti* for competitive host nodulation. *Appl. Environ. Microbiol.* **76,** 7972–7980 (2010).
34. Poysti, N. J., Loewen, E. D. M., Wang, Z. & Oresnik, I. J. *Sinorhizobium meliloti* pSymB carries genes necessary for arabinose transport and catabolism. *Microbiology* **153,** 727–736 (2007).
35. Richardson, J. S., Hynes, M. F. & Oresnik, I. J. A genetic locus necessary for rhamnose uptake and catabolism in *Rhizobium leguminosarum* bv. trifolii. *J. Bacteriol.* **186,** 8433–8442 (2004).
36. Richardson, J. S. & Oresnik, I. J. L-Rhamnose transport is sugar kinase (RhaK) dependent in *Rhizobium leguminosarum* bv. trifolii. *J. Bacteriol.* **189,** 8437–8446 (2007).
37. Raimunda, D. & Elso-Berberián, G. Functional characterization of the CDF transporter SMc02724 (SmYiiP) in *Sinorhizobium meliloti*: Roles in manganese homeostasis and nodulation. *BBA - Biomembranes* **1838,** 3203–3211 (2014).
38. Dupont, L. *et al.* The *Sinorhizobium meliloti* ABC transporter Cho Is highly specific for choline and expressed in bacteroids from *Medicago sativa* nodules. *J. Bacteriol.* **186,** 5988–5996 (2004).
39. Jebbar, M., Sohn-Bosser, L., Bremer, E., Bernard, T. & Blanco, C. Ectoine-induced proteins in *Sinorhizobium meliloti* include an ectoine ABC-type transporter involved in osmoprotection and ectoine catabolism. *J. Bacteriol.* **187,** 1293–1304 (2005).
40. Dominguez-Ferreras, A., Muñoz, S., Olivares, J., Soto, M. J. & Sanjuán, J. Role of potassium uptake systems in *Sinorhizobium meliloti* osmoadaptation and symbiotic performance. *J. Bacteriol.* **191,** 2133–2143 (2009).
41. Borisova, S. A. *et al.* Genetic and biochemical characterization of a pathway for the degradation of 2-aminoethylphosphonate in *Sinorhizobium meliloti* 1021. *Journal of Biological Chemistry* **286,** 22283–22290 (2011).
42. Gu, X., Lee, S. G. & Bar-Peled, M. Biosynthesis of UDP-xylose and UDP-arabinose in *Sinorhizobium meliloti* 1021: first characterization of a bacterial UDP-xylose synthase, and UDP-xylose 4-epimerase. *Microbiology* **157,** 260–269 (2010).
43. Goldmann, A. *et al.* Symbiotic plasmid genes essential to the catabolism of proline betaine, or stachydrine, are also required for efficient nodulation by *Rhizobium meliloti*. *FEMS Microbio Lett* **115,** 305–312 (1994).
44. Phillips, D. A. *et al.* A new genetic locus in *Sinorhizobium meliloti* is involved in stachydrine utilization. *Appl. Environ. Microbiol.* **64,** 3954–2960 (1998).
45. Sagot, B. *et al.* Osmotically induced synthesis of the dipeptide *N*-acetylglutaminylglutamine amide is mediated by a new pathway conserved among bacteria. *Proc. Natl. Acad. Sci. U.S.A.* **107,** 12652–12657 (2010).
46. de Rudder, K. E. E., Sohlenkamp, C. & Geiger, O. Plant-exuded choline is used for rhizobial membrane lipid biosynthesis by phosphatidylcholine synthase. *J. Biol. Chem.* **274,** 20011–20016 (1999).
47. Yurgel, S. N. & Kahn, M. L. *Sinorhizobium meliloti dctA* mutants with partial ability to transport dicarboxylic acids. *J. Bacteriol.* **187,** 1161–1172 (2005).
48. Dominguez-Ferreras, A., Soto, M. J., Pérez-Arnedo, R., Olivares, J. & Sanjuán, J. Importance of trehalose biosynthesis for *Sinorhizobium meliloti* osmotolerance and nodulation of alfalfa roots. *J. Bacteriol.* **191,** 7490–7499 (2009).
49. Lynch, D. *et al.* Genetic organization of the region encoding regulation, biosynthesis, and

transport of Rhizobactin 1021, a siderophore produced by *Sinorhizobium meliloti*. *J. Bacteriol.* **183,** 2576–2585 (2001).
50. Charles, T. C., Cai, G. Q. & Aneja, P. Megaplasmid and chromosomal loci for the PHB degradation pathway in *Rhizobium (Sinorhizobium) meliloti*. *Genetics* **146,** 1211–1220 (1997).
51. Wilson, J. J. & Kappler, U. Sulfite oxidation in *Sinorhizobium meliloti*. *BBA - Bioenergetics* **1787,** 1516–1525 (2009).
52. Jones, K. M., Kobayashi, H., Davies, B. W., Taga, M. E. & Walker, G. C. How rhizobial symbionts invade plants: the *Sinorhizobium–Medicago* model. *Nature Reviews Microbiology* **5,** 619–633 (2007).
53. Lerouge, P. *et al.* Symbiotic host-specificity of *Rhizobium meliloti* is determined by a sulphated and acylated glucosamine oligosaccharide signal. *Nature* **344,** 781–784 (1990).
54. Geiger, O. & López-Lara, I. M. Rhizobial acyl carrier proteins and their roles in the formation of bacterial cell-surface components that are required for the development of nitrogen-fixing root nodules on legume hosts. *FEMS Microbio Lett* **208,** 153–162 (2002).
55. Lambert, A., Osteras, M., Mandon, K., Poggi, M. C. & Le Rudulier, D. Fructose uptake in *Sinorhizobium meliloti* is mediated by a high-affinity ATP-binding cassette transport system. *J. Bacteriol.* **183,** 4709–4717 (2001).
56. White, C. E., Gavina, J. M. A., Morton, R., Britz-McKibbin, P. & Finan, T. M. Control of hydroxyproline catabolism in *Sinorhizobium meliloti*. *Mol. Microbiol.* **85,** 1133–1147 (2012).
57. Cheng, J., Poduska, B., Morton, R. A. & Finan, T. M. An ABC-type cobalt transport system is essential for growth of *Sinorhizobium meliloti* at trace metal concentrations. *J. Bacteriol.* **193,** 4405–4416 (2011).
58. Boncompagni, E. *et al.* Characterization of a *Snorhizobium meliloti* ATP-binding cassette histidine transporter also involved in betaine and proline uptake. *J. Bacteriol.* **182,** 3717–3725 (2000).
59. MacLean, A. M., Haerty, W., Golding, G. B. & Finan, T. M. The LysR-type PcaQ protein regulates expression of a protocatechuate-inducible ABC-type transport system in *Sinorhizobium meliloti*. *Microbiology* **157,** 2522–2533 (2011).
60. MacLean, A. M., MacPherson, G., Aneja, P. & Finan, T. M. Characterization of the β-ketoadipate pathway in *Sinorhizobium meliloti*. *Appl. Environ. Microbiol.* **72,** 5403–5413 (2006).
61. Yurgel, S. N., Mortimer, M. W., Rice, J. T., Humann, J. L. & Kahn, M. L. Directed construction and analysis of a *Sinorhizobium meliloti* pSymA deletion mutant library. *Appl. Environ. Microbiol.* **79,** 2081–2087 (2013).
62. Gage, D. J. & Long, S. R. α-Galactoside uptake in *Rhizobium meliloti*: isolation and characterization of *agpA*, a gene encoding a periplasmic binding protein required for melibiose and raffinose utilization. *J. Bacteriol.* **180,** 5739–5748 (1998).
63. Kanehisa, M. *et al.* Data, information, knowledge and principle: back to metabolism in KEGG. *Nucleic Acids Res.* **42,** D199–205 (2014).
64. Caspi, R. *et al.* The MetaCyc database of metabolic pathways and enzymes and the BioCyc collection of Pathway/Genome Databases. *Nucleic Acids Res.* **42,** D459–71 (2014).
65. Geddes, B. A. *et al.* A locus necessary for the transport and catabolism of erythritol in *Sinorhizobium meliloti*. *Microbiology* **156,** 2970–2981 (2010).

66. Reed, J. L., Vo, T. D., Schilling, C. H. & Palsson, B. Ø. An expanded genome-scale model of *Escherichia coli* K-12 (iJR904 GSM/GPR). *Genome Biol.* **4,** R54 (2003).
67. Schellenberger, J. *et al.* Quantitative prediction of cellular metabolism with constraint-based models: the COBRA Toolbox v2.0. *Nat Protoc* **6,** 1290–1307 (2011).
68. Yamada, T., Letunic, I., Okuda, S., Kanehisa, M. & Bork, P. iPath2.0: interactive pathway explorer. *Nucleic Acids Res.* **39,** W412–5 (2011).
69. Knee, E. M. *et al.* Root mucilage from pea and its utilization by rhizosphere bacteria as a sole carbon source. *Mol Plant Microbe Interact* **14,** 775–784 (2001).
70. Moody, S. F., Clarke, A. E. & Bacic, A. Structural analysis of secreted slimre from wheat and cowpea roots. *Phytochemistry* **27,** 2857–2861 (1988).
71. Bringhurst, R. M., Cardon, Z. G. & Gage, D. J. Galactosides in the rhizosphere: Utilization by *Sinorhizobium meliloti* and developmentof a biosensor. *Proc. Natl. Acad. Sci. U.S.A.* **98,** 4540–4548 (2001).
72. Lipton, D. S., Blanchar, R. W. & Blevins, D. G. Citrate, malate, and succinate concentration in exudates from P-sufficient and P-stressed *Medicago sativa* L. seedlings. *Plant Physiol.* **85,** 315–317 (1987).
73. Kamilova, F. *et al.* Organic acids, sugars, and L-tryprophane in esudates of vegetables growing on stonewool and their effects on activities of rhizosphere bacteria. *Mol Plant Microbe Interact* **19,** 250–256 (2006).
74. Boulter, D., Jeremy, J. J. & Wilding, M. Amino acids liberated into the culture medium by pea seedling roots. *Plant Soil* **24,** 121–127 (1966).
75. Naher, U. A., Radziah, O., Halimi, M. S., Shamsuddin, Z. H. & Mohd Razi, I. Effect of inoculation on root exudates carbon sugar and amino acids production of different rice varieties. *Research Journal of Microbiology* **3,** 580–587 (2008).
76. Murayama, S. Persistency and monosaccharide composition of polysaccharides of soil which received no plant materials for a certain period under field conditions. *Soil Science and Plant Nutrition* **27,** 463–475 (1981).
77. Jaeger, C. H., III, Lindow, S. E., Miller, W., E, C. & Firestone, M. K. Mapping of sugar and amino acid availability in soil around roots with bacterial sensors of sucrose and tryptophan. *Appl. Environ. Microbiol.* **65,** 2685–2690 (1999).
78. Jones, D. L. Organic acids in the rhizosphere – a critical review. *Plant Soil* **205,** 25–44 (1998).
79. Hertenberger, G., Zampach, P. & Bachmann, G. Plant species affect the concentration of free sugars and free amino acids in different types of soil. *J Plant Nutr Soil Sci* **165,** 557–565 (2002).
