# Supplementary material for: Metabolic modelling reveals the specialization of secondary replicons for niche adaptation in Sinorhizobium meliloti
Source: Nat Commun. 2016 Jul 22;7:12219. doi: 10.1038/ncomms12219 (PMC4961836; doi:10.1038/ncomms12219)
Supplement: Supplementary Data 2 — Contains all the raw Phenotype MicroArrayTM data that was generated in this study in the form of .csv files. [file ncomms12219-s3.zip › Biolog_curves.pdf]

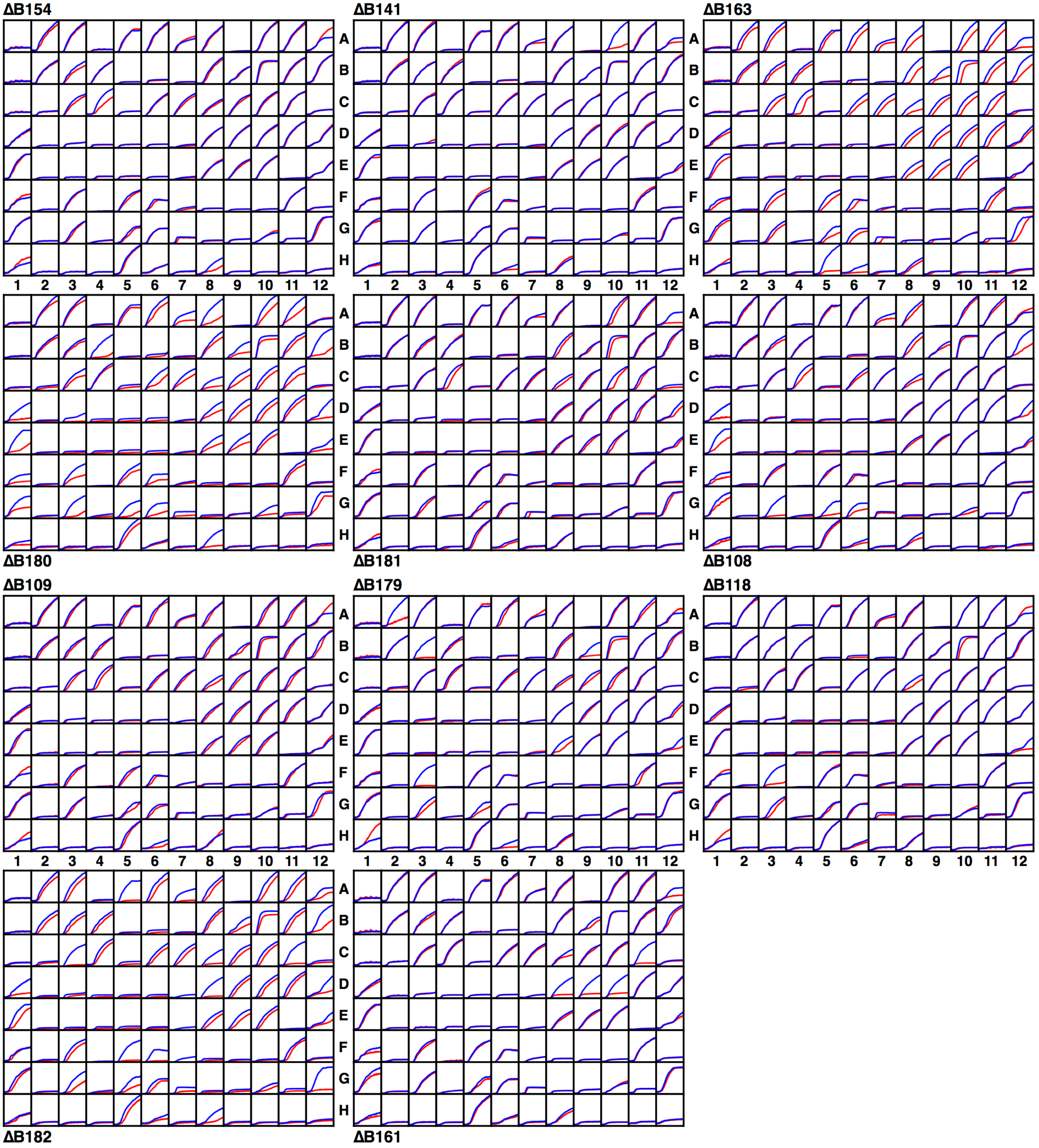

Supplementary Figure 3. Metabolic activity in the PM1 plates. Pairwise comparisons of the growth of *S. meliloti* RmP110 (blue) and the indicated deletion mutant (red) in each well of the PM1 plates. Wells are not blanked with the carbon free well (A1).

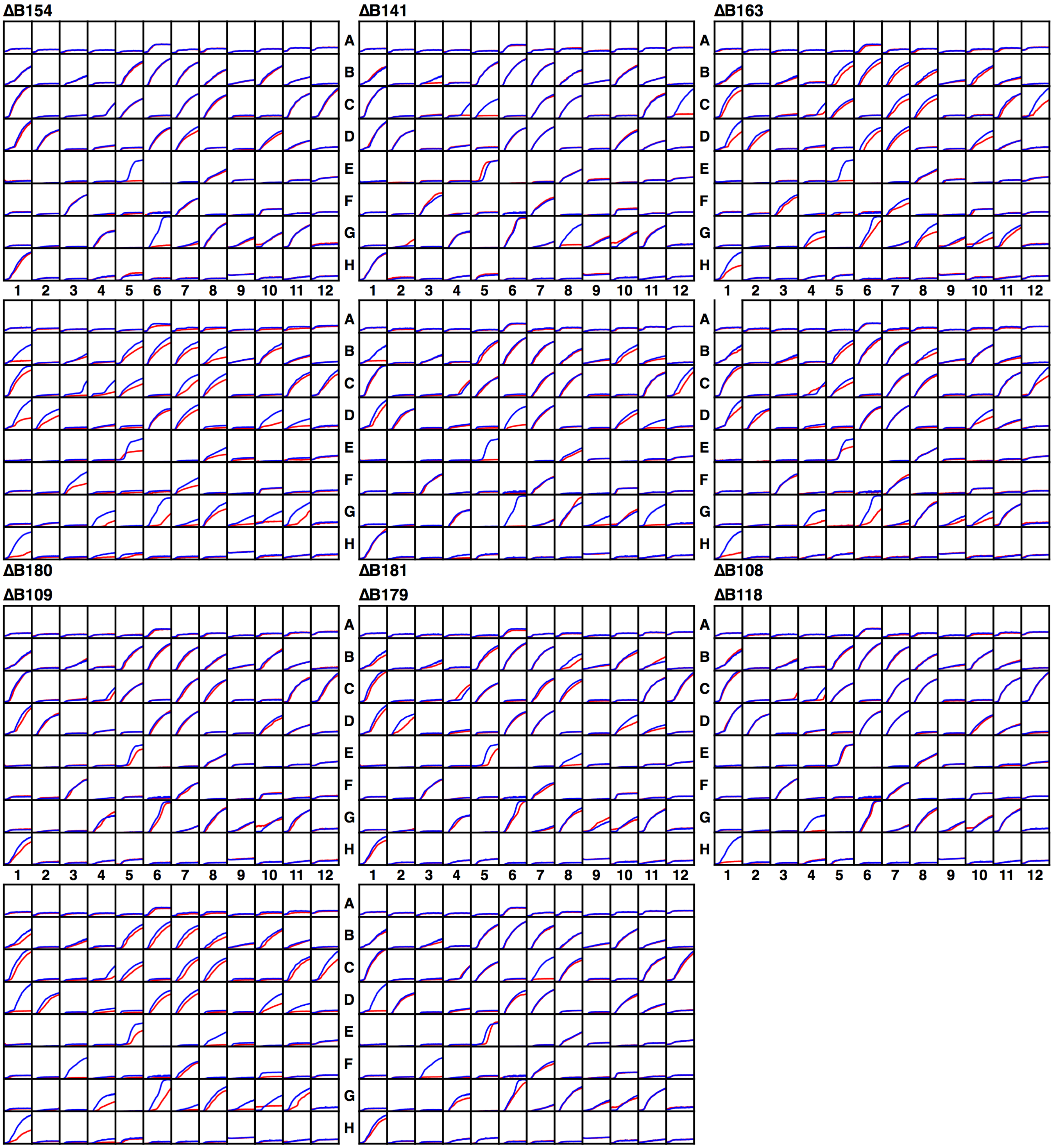

Supplementary Figure 4. Metabolic activity in the PM2A plates. Pairwise comparisons of the growth of *S. meliloti* RmP110 (blue) and the indicated deletion mutant (red) in each well of the PM2A plates. Wells are not blanked with the carbon free well (A1).
